# High Expression of EpCAM and Sox2 is a Positive Prognosticator of Clinical Outcome for Head and Neck Carcinoma

**DOI:** 10.1038/s41598-018-32178-8

**Published:** 2018-10-01

**Authors:** Philipp Baumeister, Alessandra Hollmann, Julia Kitz, Artemis Afthonidou, Florian Simon, Julius Shakhtour, Brigitte Mack, Gisela Kranz, Darko Libl, Martin Leu, Markus A. Schirmer, Martin Canis, Claus Belka, Horst Zitzelsberger, Ute Ganswindt, Julia Hess, Mark Jakob, Kristian Unger, Olivier Gires

**Affiliations:** 1 Department of Otorhinolaryngology, Head and Neck Surgery, Grosshadern Medical Center, Ludwig-Maximilians-University Munich, Marchioninistr. 15, 81377 Munich, Germany; 2 Clinical Cooperation Group “Personalized Radiotherapy in Head and Neck Cancer”, Helmholtz Zentrum, Munich, Germany; 3 Institute of Pathology, University Medical Center Göttingen, Göttingen, Germany; 4 Department of Radiation Oncology, University Medical Center Göttingen, Göttingen, Germany; 5 Department of Radiation Oncology, Ludwig-Maximilians-University Munich, Munich, Germany; 6 Research Unit Radiation Cytogenetics, Helmholtz Zentrum München, Research Center for Environmental Health (GmbH), Ingolstädter Landstraße 1 D-85764, Neuherberg, Germany

## Abstract

Locally advanced head and neck squamous cell carcinomas (HNSCC) have limited prognosis due to frequent treatment failure. Currently, TNM-classification and human papillomavirus (HPV) infection are the sole clinical prognosticators of outcome. Tumor heterogeneity and stemness based on epithelial-mesenchymal-transition reportedly associate with therapy resistance. The capacity of epithelial marker EpCAM (EpEX), stemness regulator Sox2 and mesenchymal marker vimentin to predict clinical outcome of HSNCC patients was assessed upon immunohistochemistry staining in two cohorts of HNSCC patients treated with surgery and adjuvant radio (chemo) therapy (n = 94) and primary radio (chemo) therapy (n = 94), respectively. Prognostic values with respect to overall, disease-free and disease-specific survival were assessed in uni- and multivariate cox proportional hazard models to generate integrated risk scores. EpEX, Sox2 and vimentin displayed substantial inter- and intratumoral heterogeneity. EpEX^high^ and Sox2^high^ predicted improved clinical outcome in the discovery cohort and in the HPV-negative sub-cohort. EpEX^high^ and Sox2^high^ were confirmed as prognosticators of clinical outcome in the validation cohort treated with definitive radio(chemo)therapy. Importantly, EpEX^high^ identified patients with improved survival within the HPV-negative subgroup of the validation cohort. Hence, Sox2^high^ and particularly EpEX^high^ have potential as tools to predict clinical performance of HNSCC patients, foremost HPV-negative cases, in the frame of molecular-guided treatment decision-making.

## Introduction

Head and neck squamous cell carcinomas (HNSCC) are typically induced by carcinogenic components of alcohol and tobacco or -primarily for oropharyngeal carcinomas- through infection with high-risk human papillomaviruses (HPV)^1,2^. Currently, three separate HPV-negative and further two biologically differing HPV-positive subtypes of HSNCC can be distinguished^2^. Despite aggressive treatment, HNSCC display dismal 5-year overall survival (OS) and disease-free survival (DFS) rates of less than 45%^3^. The clinical outcome including OS and DFS strongly depends on tumor size, locoregional spread, resection margins, extracapsular extension (ECE), lymphovascular invasion and systemic dissemination^1^. Accordingly, TNM classification is an accepted prognostic factor for HNSCC that can serve to predict survival probabilities, but does not allow for a more differential, adjusted, and personalized prediction of the outcome of tumor patients with similar TNM status. More subtle differences across patients that might impact on outcome are rather neglected by merely focusing on TNM classification, hence the need for valid biomarkers. HPV-status is currently the strongest available marker identifying HPV-positive HNSCC as distinct subgroup of HNSCC with prolonged OS, which is meanwhile implemented in a new TNM classification system for p16-positive oropharyngeal carcinomas^4^.

Owing to a long-lasting tobacco and nicotine abuse, frequently in conjunction with alcohol abuse, and to the high mutagenic potential of these prime risk factors associated with HNSCC, the mutational burden that eventually contributes to cellular heterogeneity is very high with an average of 130 mutations per tumor^5,6^. Heterogeneity contributes to the emergence of aggressive and treatment-resistant cell subsets through pre-existing subpopulations in primary tumors^7^. Phenotypic changes along the epithelial-to-mesenchymal transition (EMT/MET) allow carcinoma cells to gradually and reversibly change phenotype from adhesive, stationary and proliferative cells (epithelial) to migratory, invasive and rather resting cells (mesenchymal)^8–11^. EMT is reported to increase cellular stemness, provide invasive features required for tumor progression, and enhance treatment resistance^9,11,12^. Accordingly, tumor-initiating cells in HNSCC can adopt either an epithelial/proliferative (E-type) or a mesenchymal/migratory (M-type) phenotype, where M-type cells within HNSCC cell lines and primary tumors bear enhanced therapeutic resistance^13,14^. However, a strict reliance of carcinoma cells on EMT to progress through the metastatic cascade and become treatment-resistant has been challenged in some entities such as pancreatic and lung cancers^15,16^. Accordingly, the central role of EMT in cancer progression has been intensely and controversially discussed in recent years^9,17,18^.

In the present study, we analyzed the expression levels and patterns of EpCAM, Sox2 and vimentin in primary HNSCC for association with patient outcome. EpCAM is an epithelial cell marker involved in regulation of proliferation and stem cell differentiation^19–22^. EpCAM down-regulation in mesodermally differentiating embryonic stem cells that undergo EMT is an early and necessary event in gastrulation^22^, which is likewise observed in partial EMT occurring in cancer cells^23–28^. Sex-determining region Y-box 2 (Sox2) is a transcription factor required for stem cell pluripotency and reprograming of somatic cells to induced pluripotent stem cells^29^. Owing to amplification on chromosome 3q26, Sox2 is frequently over-expressed in carcinomas including HNSCC^30^. Vimentin is an intermediate filament expressed by mesenchymal cells and carcinoma cells that have undergone EMT, which is involved in the regulation of cell motility and invasion^31^.

On the basis of protein expression patterns, we have defined high expression of Sox2 (Sox2^high^) and of EpCAM (measured using antibodies against the extracellular domain EpEX; EpEX^high^) as prognostic factors for improved clinical outcome of HNSCC. EpEX^high^ was a significant prognostic factor for overall, disease-free and disease-specific survival in HPV-negative patients of two independent cohorts with fundamentally different clinical treatment. Hence, Sox2^high^ and particularly EpEX^high^ represent promising tools for improved stratification of HNSCC and, thus, for alternative therapeutic decision-making for patients with similar TNM status.

## Results

### EpEX, Sox2 and vimentin expression in the discovery HNSCC LMU cohort

Expression of epithelial marker EpCAM (detected with antibodies specific for the extracellular domain EpEX), transcription factor Sox2 and mesenchymal marker vimentin was evaluated in consecutive kryo-conserved serial sections (5 µm) of biospecimens of the retrospective LMU cohort (Table 1, upper part, and Supplementary Table 1). EpCAM and vimentin mRNA levels are major determinants of epithelial differentiation and partial EMT in HNSCC, as was recently reported using single cell RNA-sequencing data^32^. Therefore, EpCAM and vimentin proteins represent a pair of potential markers for EMT in HNSCC, which should have opposing expression patterns in tumors.

**Table 1:** Clinical parameters of HNSCC LMU (upper) and Göttingen (lower) cohorts (each n = 94) including gender, age, p16 expression, TNM status, smoking habits, and tumor sub-localization.

Variable LMU cohort					EpEx p-value	Sox2 p-value	Vimentin p-value
Gender	Male	Female					
%	78.7	21.3			0.553	0.785	0.607
Age	<50	50-69	≥70				
%	16.0	63.8	20.2		0.528	0.201	0.699
P16	Negative	Positive	n.p.		Negative < Positive	Negative < Positive	
%	51.1	30.9	18.1		0.007	0.020	0.725
T-Stage	pT1-2	pT3-4	pTx				
%	54.3	43.6	2.1		0.235	0.712	0.309
N-Stage	N0	N+	Nx			N0 < N+	
%	35.1	63.8	1.1		0.113	0.006	0.498
M-Stage	cM0	cM+	cMx				
%	98.9	1.1	0		0.667	0.851	0.430
P-Stage	Pn0	Pn1	Pnx			Pn0 < Pn1	
%	48.9	14.9	36.2		0.078	0.015	0.803
Smoking Status	Never	Former	Current	n.p			
%	14.9	25.5	47.9	11.7	0.419	0.317	0.882
Localization	Oral Cavity	Oropharynx	Hypopharynx & Larynx		OC < OP/HP/Larynx		
%	26.6	57.4	16.0		0.001	0.305	0.137
**Variable Göttingen cohort**					**EpEx** **p-value**	**Sox2** **p-value**	**Vimentin** **p-value**
Gender	Male	Female					
%	83.0	17.0			0.369	0.888	0.639
Age	<50	50-69	≥70				
%	21.3	67.0	11.7		0.508	0.206	0.11
P16	Negative	Positive					
%	52.1	47.9			0.459	0.107	0.556
T-Stage	pT1-2	pT3-4					
%	12.8	87.2			0.154	0.747	0.414
N-Stage	N0	N+			N0 < N+		
%	17.0	83.0			0.008	0.116	0.294
M-Stage	cM0	cM+					
%	89.4	10.6			0.591	0.690	0.403
Smoking Status	Never	Ever					
%	19.1	80.9			0.512	0.854	0.360
Localization	Oral Cavity	Oropharynx	Hypopharynx & Larynx			OC < OP/HP/Larynx	
%	31.9	40.4	27.7		0.135	0.004	0.814

One way ANOVA (Kruskal-Wallis-Trial) was used to compare variables and biomarkers (EpEX, Sox2, Vimentin). Antigens differentially expressed at p < 0.05 are marked with the compared groups of variables and the orientation of differential expression (low “<” high) mentioned. OC = oral cavity, OP = oropharynx carcinoma, HP = hypopharynx carcinoma.

**Figure 1:**
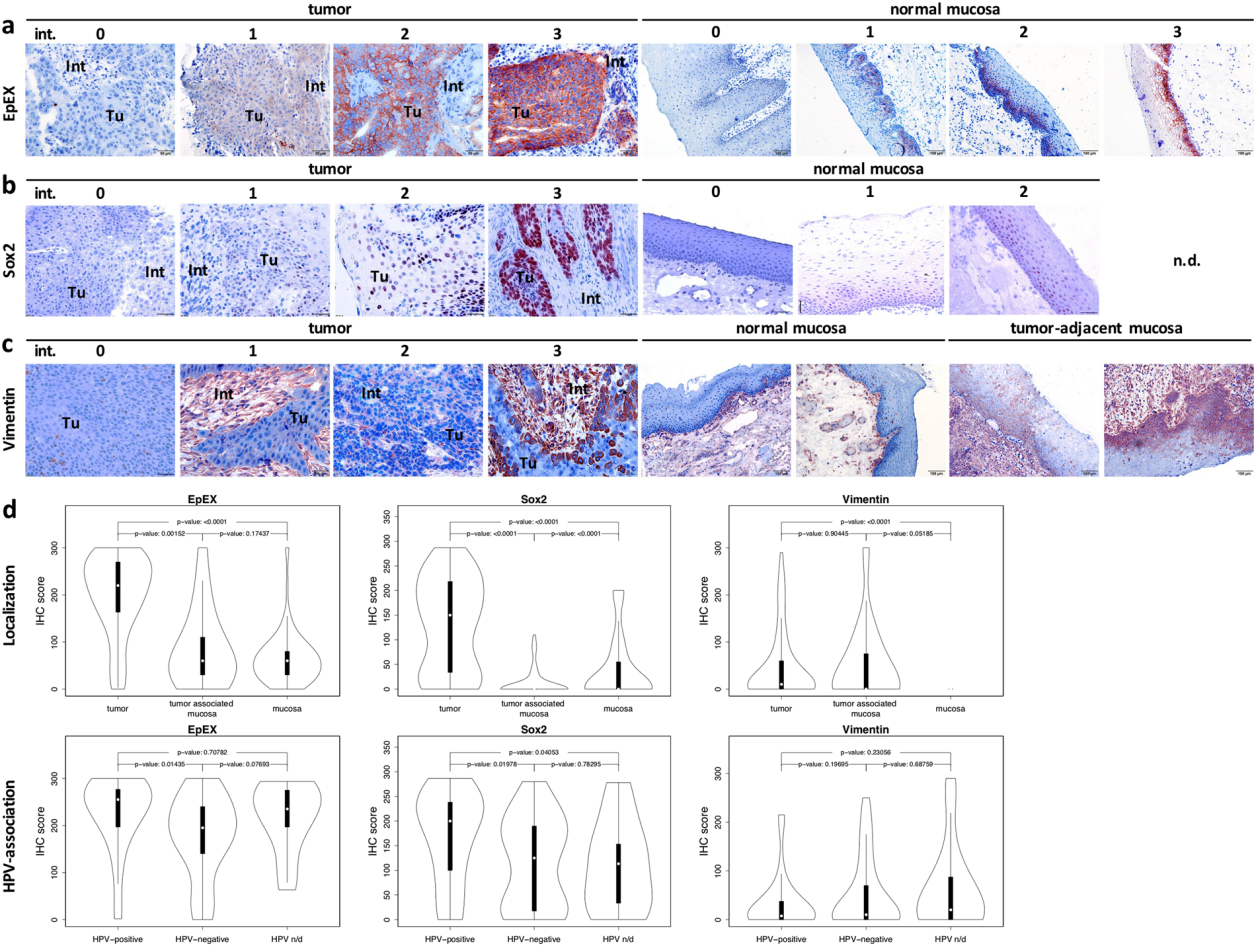
EpEX, Sox2 and vimentin expression in HNSCC and normal mucosas. (**a–c**) Shown are examples of EpEX (**a**), Sox2 (**b**) and vimentin (**c**) expression ranging from no (0), weak (1), intermediate (2) to strong (3) in HNSCC (upper panels; 200×), normal mucosas and tumor-adjacent mucosas (lower panels; 100×). EpCAM, Sox2 and vimentin staining is red-brown, nuclei and cytoplasm are counter-stained with hemalaun (blue). (n/d.: not detected). Tu: tumor; Int: interstitium. (**d**) Upper: IHC scores of EpEX, Sox2 and vimentin expression in normal mucosas, tumor-adjacent mucosas and HNSCC are displayed as violin plots including median (open circle), 1^st^ and 3^rd^ interquartile range, minima and maxima, and kernel density estimation. p-values are derived from unpaired Mann-Whitney testing. Lower: IHC scores of EpEX, Sox2 and vimentin expression in HPV-positive and –negative HNSCC are displayed as violin plots including median (open circle), 1^st^ and 3^rd^ interquartile range, minima and maxima, and kernel density estimation (n/d: not determined). P-values are derived from paired Student´s T-test following confirmation of normal distribution of data (Levene test).

Vimentin-expressing cells in the interstitium and infiltrating non-tumor cells were excluded from the analyses based on their morphology combined with intense and homogeneous antigen expression. EpEX, Sox2 and vimentin were heterogeneously expressed in tumors, ranging from no expression (0), weak (1+), intermediate (2+), to strong expression (3+) and in normal mucosa (Fig. 1a–c). In normal mucosa, EpEX and Sox2 were expressed in cells of the *stratum basale* and *stratum parabasale* only, and Sox2 expression did not exceed an intermediate (2+) level (Fig. 1a,b). Vimentin expression in normal mucosa was restricted to infiltrating mesenchymal cells and was up-regulated in tumor-adjacent mucosa with hyper- and/or dysplastic cells (Fig. 1c).

IHC scores, representing intensity and frequency of antigen expression from two to four sections of biospecimen, ranged from 0–300 with various combinations of intensities and percentages for all three antigens (Supplementary Table 1). Comparison of normal mucosa, tumor-adjacent mucosa, and tumors disclosed a strong and highly significant (p < 0.0001) up-regulation of EpEX in tumors with median IHC scores of 220 versus 60 and 50 in tumor-adjacent and healthy mucosa, respectively (Fig. 1d). Sox2 expression was strongly and highly significantly (p < 0.0001) up-regulated in tumors versus normal and tumor-adjacent mucosa (median IHC score of 150 in tumors versus 0 in normal and tumor-adjacent mucosa), whereas vimentin expression showed a moderate induction in tumor-adjacent mucosa and tumors as compared to normal mucosa (p < 0.0001) (Fig. 1d).

An association of EpEX and Sox2 IHC scores with HPV-status was observed, where increased expression levels were shown for both in HPV-positive HNSCC (Table 1 and Fig. 1d). Vimentin did not show any HPV-associated difference in expression (Fig. 1d).

### EpEX, Sox2 and vimentin expression patterns in HNSCC

**Figure 2:**
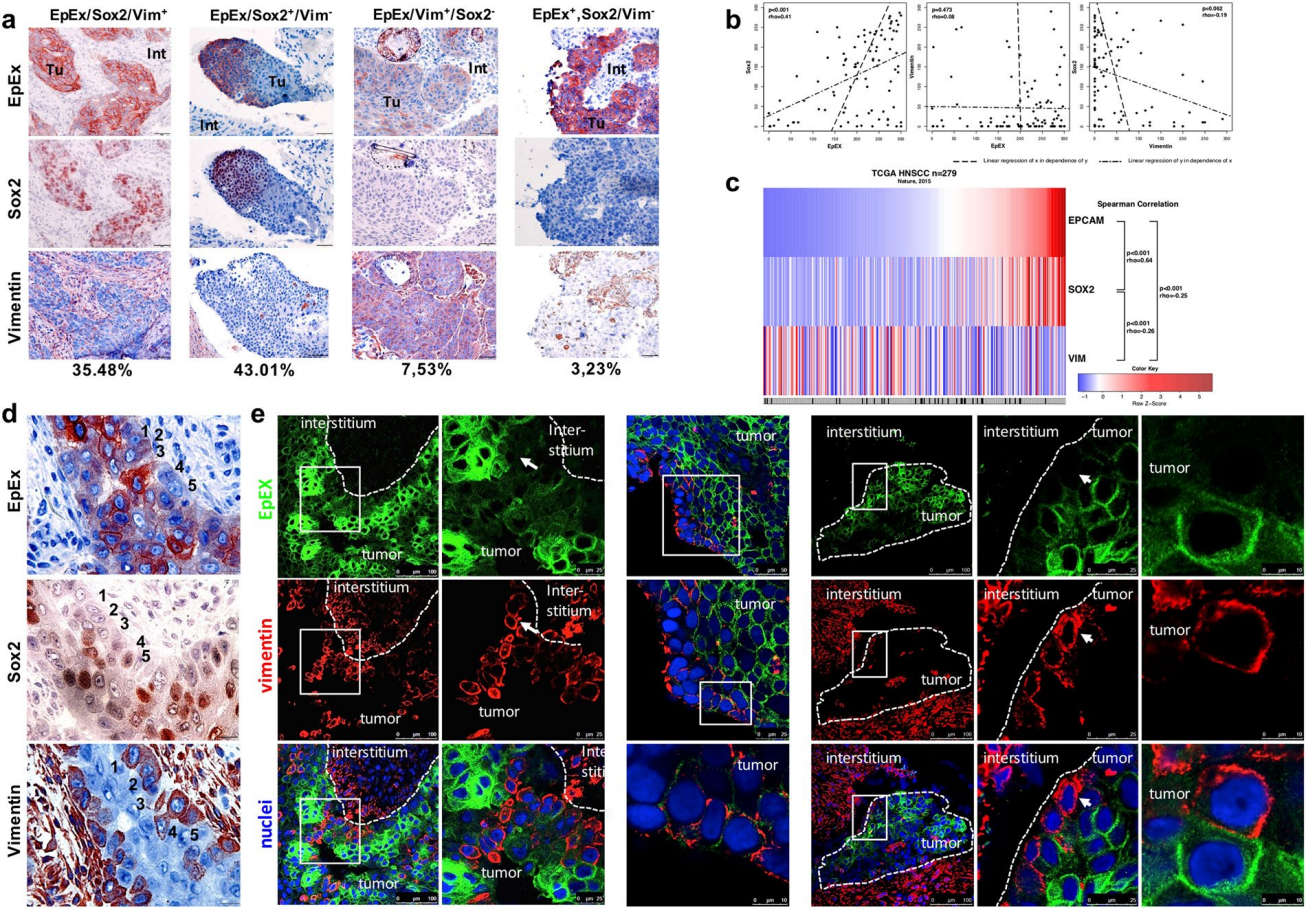
EpEX, Sox2 and vimentin expression patterns in HNSCC. (**a**) Examples of the major four expression patterns of EpEX, Sox2 and vimentin are shown with according frequencies in percentages. All antigen-specific staining (200×) in consecutive sections are depicted in red-brown, while nuclei and cytoplasm are counter-stained with hemalaun (blue). Tu: tumor; Int: interstitium. (**b**) IHC scores of EpEX, Sox2 and vimentin for HNSCC (n = 94) were plotted and displayed with linear regression curves, p-values and Spearman’s rank rho coefficient. (**c**) mRNA expression level z-scores for EpCAM, Sox2 and vimentin from The Cancer Genome Atlas (TCGA) HNSCC cohort (n = 279) were downloaded at cBioPortal and were depicted as an expression heat-map. Spearman correlation analysis results with rho coefficient and p-values are indicated. (**d**) EpEX, Sox2 and vimentin expression in serial sections of HNSCC is depicted (400×). Examples of tumor cells with reduced EpCAM and increased vimentin expression are numbered (1 to 5). (**e**) Simultaneous immunofluorescence staining of EpEX and vimentin are visualized in confocal laser scanning microscopy for EpEX (green), vimentin (red) and as a merged image (combined colors). Nuclear DNA is stained in blue with DAPI. The margin between tumor and interstitium, and enlarged areas are marked as doted lines and solid squares. Cells with loss of EpCAM and gain of vimentin expression are marked with white arrows. Left panel: Depicts the edge of a tumor area; center panel: depicts vimentin-positive tumor cells at the edge of a tumor area; Right panel: depicts a small tumor island surrounded by interstitium, and rightmost panel: depicts two carcinoma cells demonstrating the co-localization of EpCAM^+^/vimentin^−^ and EpCAM^−^/vimentin^+^ cells at the border to the interstitium.

Two major and two minor tumor expression patterns of EpEX, Sox2 and vimentin were observed in the LMU cohort: High expression of EpCAM and Sox2, but lack of vimentin expression (EpEX/Sox2^+^/vimentin^−^ 43.01%), high expression of all three antigens (EpEX/Sox2/vimentin^+^ 35.48%), high expression of EpCAM and vimentin, but lack of Sox2 expression (EpEX/vimentin^+^/Sox2^−^ 7.53%) and high expression of EpCAM, but lack of Sox2 and vimentin expression (EpEX^+^/Sox2/vimentin^−^ 3.23%). Representative staining of all four major expression patterns on consecutive sections of tumor samples are shown in Fig. 2a. Significant positive correlation of Sox2 with EpEX (Fig. 2b; rho = 0.41, p-value < 0.001) and a trend for a weak, negative correlation of Sox2 with vimentin was found (Fig. 2b; rho = −0.19, p-value = 0.062). Hence, epithelial marker EpCAM showed co-expression with reprogramming factor Sox2, but mesenchymal marker vimentin was independent from EpCAM expression and its expression negatively related to that of Sox2.

In order to further study the correlation of EpCAM, Sox2 and vimentin at the transcriptional level, mRNA expression data from HNSCC patients (n = 279) included in The Cancer Genome Atlas (TCGA) cohort were analyzed^5^. TCGA data confirmed a strong positive and significant correlation of EpCAM and Sox2 expression (rho = 0.64, p < 0.001). Furthermore, EpCAM and Sox2 negatively correlated with vimentin expression with rho = −0.25 (EpCAM, p < 0.001) and rho = −0.26 (Sox2, p < 0.001) values, respectively (Fig. 2c). Stratification according to the HPV-status (HPV-negative = 243 cases; HPV-positive = 36 cases) disclosed no correlation of EpCAM mRNA expression with HPV infection, whereas Sox2 and vimentin correlated positively (Mann-Whitney p-value = 7.9E-05) and negatively (Mann-Whitney p-value = 0.04008), respectively. Correlation of antigen expressions were only addressed in HPV-negative samples, owing to the limited number of HPV-positive specimen. EpCAM and Sox2 positively correlated (rho = 0.633, p-value < 2.2E-16), EpCAM and vimentin negatively correlated (rho = −0.267, p-value = 2.5E-05), and Sox2 and vimentin negatively correlated (rho = −0.25, p-value = 7.69E-05). Stratification of patients within the TCGA cohort according to tumor size disclosed higher expression of EpCAM expression in bigger tumors (T-classification 3–4 versus 1–2) (Mann-Whitney p-value = 0.013), whereas Sox2 and vimentin expression did not correlate with tumor size. No correlation was observed between the three antigens and tumor grade of differentiation. In summary, expression patterns of EpCAM and Sox2 positively correlated at the mRNA level in a large independent HNSCC patient cohort, whereas vimentin showed an inverse expression pattern compared to that of EpCAM and Sox2.

Heterogeneous expression patterns of EpEX, Sox2 and vimentin were analyzed in further detail at the single cell level using immunohistochemistry and immunofluorescence staining. Frequent co-expression and simultaneous lack of EpEX and Sox2 was confirmed (Fig. 2d). Mutually exclusive expression of EpEX and vimentin was recurrently observed at the interface of the tumor area to interstitium. EpCAM^low/-^/vimentin^+^ cells lined up as agglomerates within tumor protrusions (Fig. 2d,e left panel) and as islands of vimentin^+^ cells at the edge of large tumor areas (Fig. 2e, central panel). Small islands of tumor cells separated from the major tumor area frequently showed loss of EpCAM expression at the edges, which was linked to gain of vimentin expression (Fig. 2e, right panel), itself suggestive of partial EMT within tumors. EMT-related phenotypes co-existed in HNSCC samples with single cells displaying exclusive expression of epithelial marker EpCAM adjacent to cells characterized by complete lack of EpCAM and gain of mesenchymal marker vimentin (Fig. 2e, most right panel). Such differences in intratumoral sub-localization of antigen expression is in accordance with reports from Puram *et al*.^32^, who reported on a predominant partial EMT at the leading edges of tumors. These might represent tumor cells with increased capacity of delamination and local invasion of surrounding stromal tissue, and are thus of potential clinical importance.

In order to exclude that vimentin^+^ cells in tumor areas represented infiltrating non-tumor cells, selected tumor specimens were stained with vimentin- and either CD31- (endothelial cells), CD68- (monocytes/macrophages), or CD90-specific antibodies (fibroblasts), respectively. Vimentin^+^ cells did neither express CD31 nor CD90, but vimentin/CD68^+^ macrophages/monocytes were detected (Supplementary Fig. 1). However, vimentin^+^ tumor cells were morphologically distinguishable from infiltrating, vimentin-expressing monocytes/macrophages.

### Association of EpEX, Sox2 and vimentin with clinical outcome

**Figure 3:**
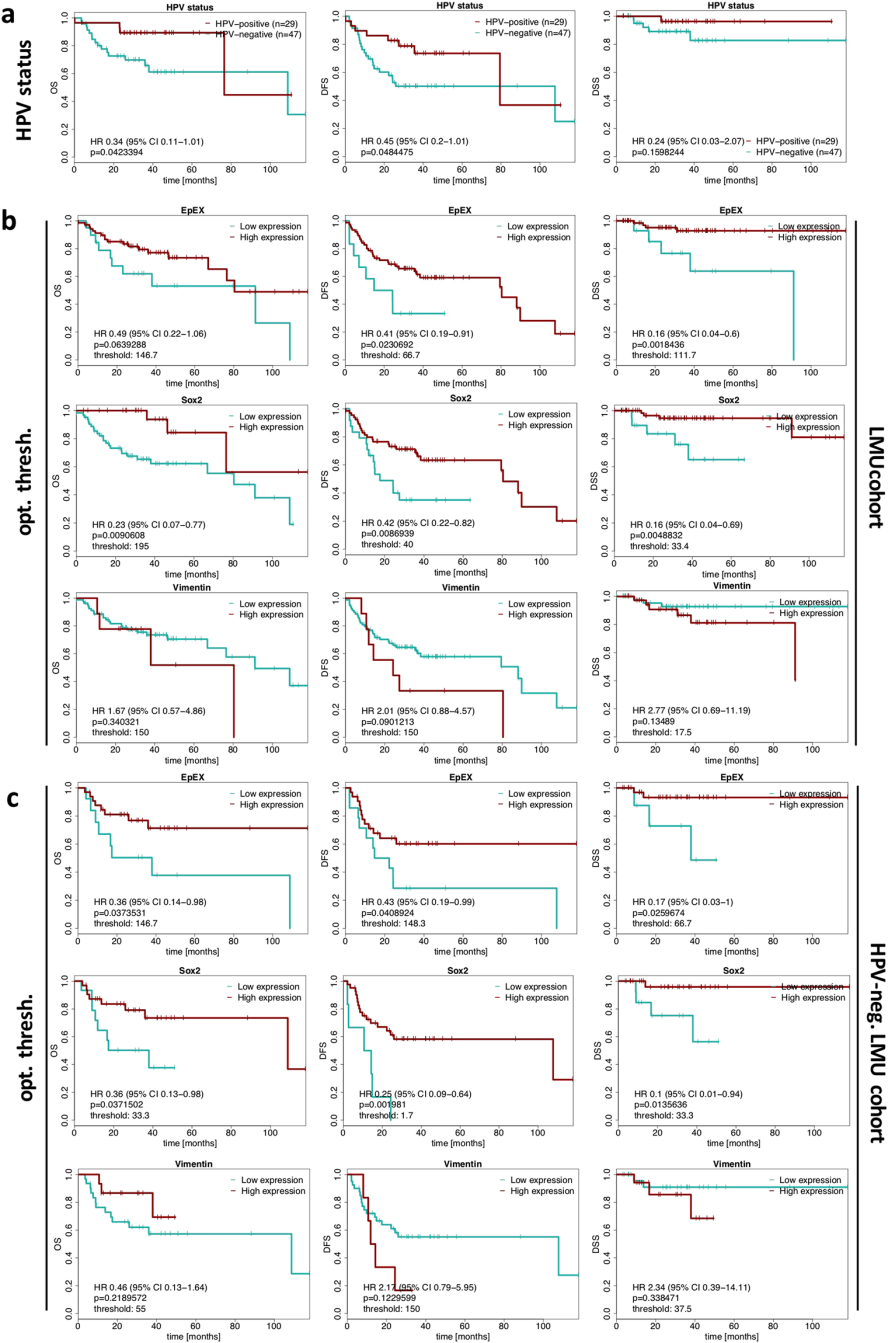
Association of single EpEX, Sox2 and vimentin IHC scores with clinical outcome in the LMU cohort. (**a**) Kaplan-Meier curves for the endpoints overall survival (OS), disease-free survival (DFS) and disease-specific survival (DSS) in HNSCC patients stratified according to HPV-status (HPV-positive versus HPV-negative). Hazard ratios (HR) with 95% confidence intervals (CI) and p-values (log-rank test) are indicated. (**b**,**c**) Kaplan-Meier curves for overall survival (OS), disease-free survival (DFS) and disease-specific survival (DSS) in HNSCC patients stratified into groups with low and high expression of EpEX, Sox2 and vimentin. Hazard ratios (HR) with 95% confidence intervals (CI), p-values (log-rank test) and the applied optimized threshold for the classification of patient subgroups are indicated for the entire cohort (**b**) and the HPV-negative sub-cohort (**c**).

IHC scores for each single antigen were analyzed for association with clinical endpoints, in order to define potential prognostic factors. Stratification according to HPV-status confirmed improved OS and DFS for HPV^+^ patients, while no statistical difference was observed with respect to DSS (Fig. 3a). Threshold optimization was applied to IHC scores of all antigens to define optimal cut-offs for each clinical endpoint. Accordingly, patients were separated into groups of antigen^high^ and antigen^low^ expressors, *i*.*e*. if their antigen expression was above or below calculated thresholds, respectively. Optimized EpEX IHC score thresholds that maximized splits between high- and low expressors were thresholds of 146.7, 66.7, and 111.7 for OS, DFS, and DSS hazard ratios, respectively (Fig. 3b). Optimized IHC score thresholds for Sox2 were 195, 40, and 33.4 for OS, DFS, and DSS hazard ratios, respectively (Fig. 3b) and that of vimentin were 150, 150, and 17.5 for OS, DFS, and DSS hazard ratios, respectively (Fig. 3b). In the LMU cohort (n = 94) irrespective of the HPV-status, EpEX^high^ patients showed significantly improved DFS and DSS rates, and a strong tendency towards improved OS. Significantly enhanced OS, DFS, and DSS were observed for Sox2^high^ classified patients (Fig. 3b). Vimentin^low^ classified patients showed a trend towards improved clinical outcome (Fig. 3b). Since EpEX and Sox2 expression levels were associated with HPV-status, which is known as strong prognostic factor in HNSCC, we further assessed the association of EpEX, Sox2 and vimentin in the subgroup of HPV-negative patients of the LMU cohort (n = 47). Both, EpEX^high^ and Sox2^high^ were significantly associated with enhanced OS, DFS, and DSS in HPV-negative patients (Fig. 3c).

### Validation of the prognostic value of EpEx, Sox2 and vimentin

In order to independently analyze the prognostic value of EpEX, Sox2 and vimentin, expression levels of all three antigens were analyzed in an additional cohort of HNSCC that reflects the second major treatment regimen for HNSCC. The Göttingen validation cohort is composed of HNSCC samples (n = 94) from patients treated with definitive radio(chemo)therapy without prior surgery owing to the presence of distant metastases already at initial diagnosis or due to unresectable tumors with respect to localization and size. Accordingly, patients within the Göttingen cohort had significantly more advanced tumors than patients in the LMU cohort (pT3–4 87.2% versus 43.6%; Chi-square p-value < 2 × 10^−9^), significantly more frequently locoregional lymph node metastases (pN + 83.0% versus 63.8%; Chi-square p-value < 0.01), significantly more often distant metastases (pM + 10.6% versus 1.1%; Chi-square p-value 0.01), and significantly differed in localization (Chi-square p-value 0.04), but did not significantly differ in their HPV-status (Tables 1 and 2). Taken together, these unfavorable clinical parameters resulted in worse outcome of patients in the Göttingen cohort. Expression of EpEX, Sox2, and vimentin was assessed in two to four punches per biospecimen on tissue micro-arrays of the validation cohort. Given the lack of primary surgery, influence of all three antigens on clinical endpoints in the presence of primary tumors can be assessed.

**Table 2:** Statistical analysis of clinical parameters of HNSCC LMU and Göttingen cohorts (each n = 94) including gender, age, p16 expression, TNM status, smoking habits, and tumor sub-localization.

Variable	Cohort	Categories			P-value
Sex		Male	Female		
	Göttingen	78 (83%)	16 (17%)		
	LMU	74 (78.7%)	20 (21.3%)		0,58
Age		<50	50-69	≥70	
	Göttingen	20 (21.3%)	63 (67%)	11 (11.7%)	
	LMU	15 (16%)	60 (63.8%)	19 (20.2%)	0,23
P16		negative	positive		
	Göttingen	49 (52.1%)	45 (47.9%)		
	LMU	48 (62.3%)	29 (37.7%)		0,24
T-stage		pT1-2	pT3-4	pTx	
	Göttingen	12 (12.8%)	82 (87.2%)	0 (0%)	
	LMU	51 (54.3%)	41 (43.6%)	2 (2.1%)	<2 × 10^−9^
N-stage		N0	N+	Nx	
	Göttingen	16 (17%)	78 (83%)	0 (17%)	
	LMU	1 (35.1%)	33 (63.8%)	60 (1.1%)	<0.01
M-Stage		cM0	cM+		
	Göttingen	93 (98.9%)	1 (1.1%)		
	LMU	84 (89.4%)	10 (10.6%)		0,01
Smoking		Never	Ever		
	Göttingen	18 (19.1%)	76 (80.9%)		
	LMU	14 (16.9%)	69 (83.1%)		0,84
Localization		Oral Cavity	Oropharynx	Hypopharnyx/Larynx	
	Göttingen	30 (31.9%)	38 (40.4%)	26 (27.7%)	
	LMU	25 (26.6%)	54 (57.4%)	15 (16%)	0,04

Chi-square test was applied to calculate p-values.

**Figure 4:**
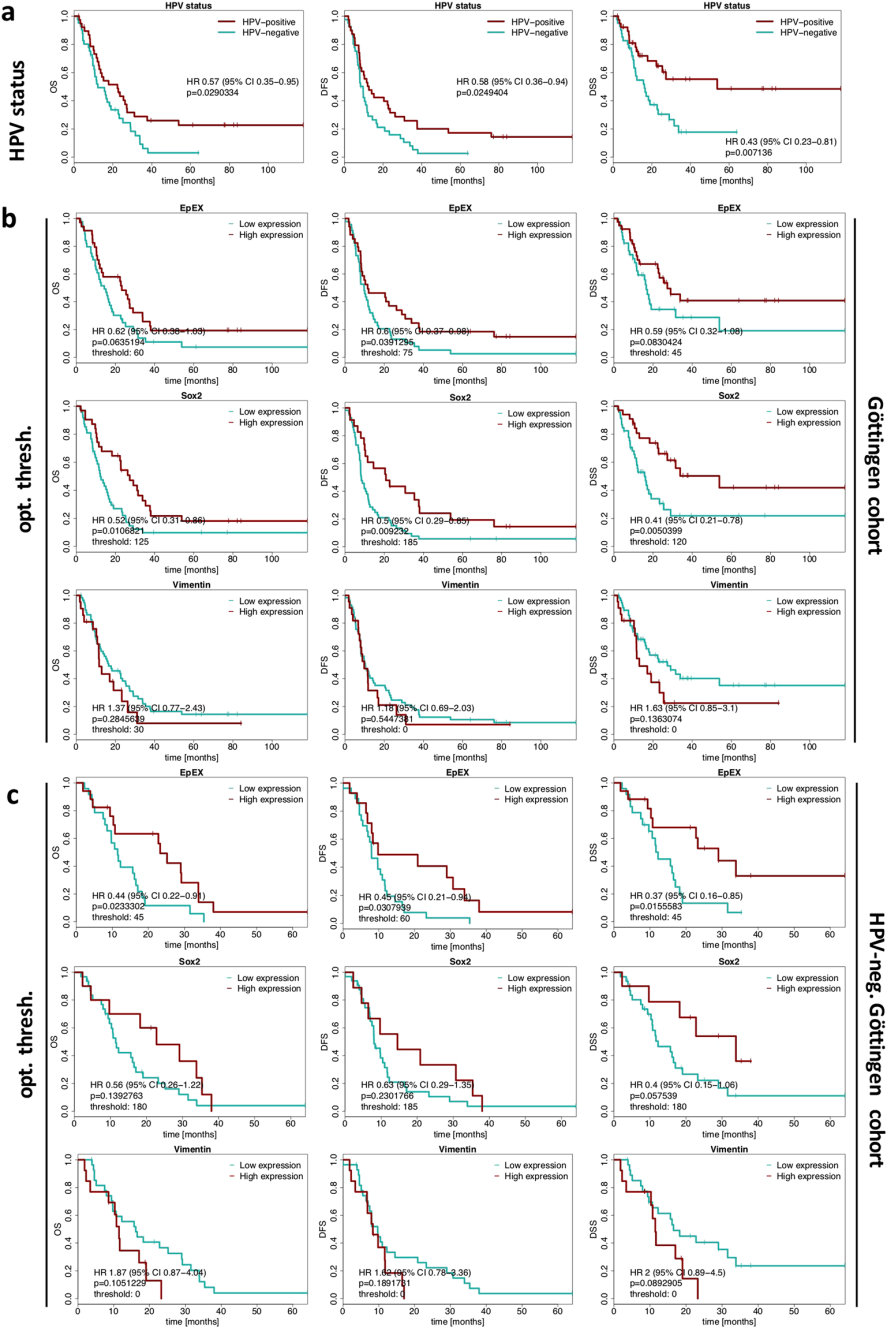
Association of single EpEX, Sox2 and vimentin IHC scores with clinical outcome in the Göttingen cohort. (**a**) Kaplan-Meier curves for the endpoints overall survival (OS), disease-free survival (DFS) and disease-specific survival (DSS) in HNSCC patients stratified according to HPV-status (HPV-positive versus HPV-negative). Hazard ratios (HR) with 95% confidence intervals (CI) and p-values (log-rank test) are indicated. (**b**,**c**) Kaplan-Meier curves for overall survival (OS), disease-free survival (DFS) and disease-specific survival (DSS) in HNSCC patients stratified into groups with low and high expression of EpEX, Sox2 and vimentin. Hazard ratios (HR) with 95% confidence intervals (CI), p-values (log-rank test) and the applied optimized threshold for the classification of patient subgroups are indicated for the entire cohort (**b**) and the HPV-negative sub-cohort (**c**).

HPV-status was a prognostic factor for all clinical endpoints analyzed within the Göttingen cohort (Fig. 4a). Patients classified as EpEX^high^ showed significantly enhanced DFS and a trend towards improved OS and DSS. Patients classified as Sox2^high^ showed significantly enhanced OS, DFS, and DSS (Fig. 4b). Vimentin^low^ classified patients showed a tendency of slightly improved outcome (OS and DSS; Fig. 4b). EpEX^high^, but not Sox2^high^, was a prognosticator of significantly improved OS, DFS, and DSS in the subgroup of HPV-negative HNSCC patients within the Göttingen cohort (Fig. 4c). Sox2^high^ HPV-negative patients showed a strong trend towards enhanced DSS (p = 0.057) (Fig. 4c). Thus, EpEX^high^ distinguished HPV-negative patients with improved clinical outcome in the discovery and validation cohorts.

### EpEX- and Sox2-based risk factor for the prediction of HNSCC clinical outcome

Since EpEX and Sox2 expression were prognostic in univariate analyses, we integrated measurements on both proteins in one multivariate cox proportional hazard model in order to calculate an integrated risk score for each patient. The cox model was fitted in the LMU cohort data (discovery cohort) followed by calculation of the risk scores and a median threshold, allowing assortment of patients into high- and low-risk. The fit coefficients and the threshold determined in the discovery cohort were used in combination with EpEX and Sox2 expressions measured in the validation cohort (Göttingen cohort) to calculate risk scores for subsequent assignment of patients of the Göttingen cohort to risk groups.

**Figure 5:**
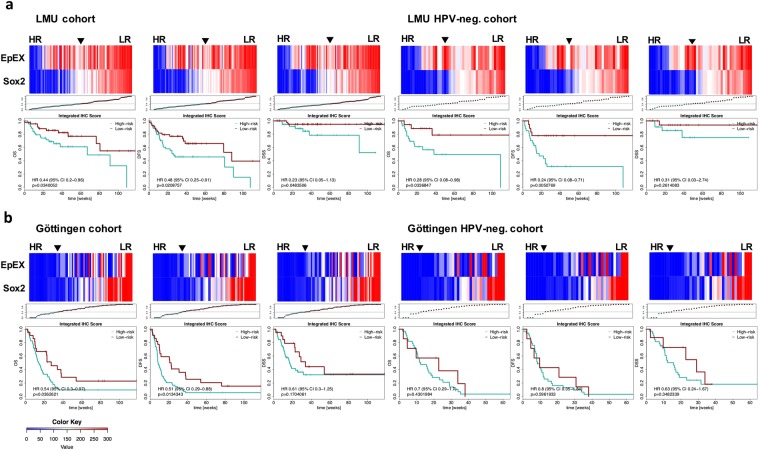
Risk factor as a prognosticator for HNSCC. (**a**,**b**) Optimized thresholds for Sox2 and EpEX IHC scores were implemented in an integrated risk factor to predict the clinical outcome of HSNCCs. Shown is a heat-map representation of the composition of the risk factor across HNSCC patients of the LMU cohort (**a**; n = 94) and the Göttingen cohort (**b**; n = 94). Additionally, both cohorts were subdivided into HPV-negative sub-cohorts (right panels). Stratification of patients was conducted according to the SEV-factor into high and low scores. Overall survival (OS), disease-free survival (DFS) and disease-specific survival (DSS) are depicted as Kaplan-Meier curves with hazard ratio (HR) at indicated 95% confidence interval (CI) and log-rank test p-values.

IHC scores of EpEX and Sox2 in relation to the risk score and Kaplan Meier plots are depicted for OS, DFS and DSS of the LMU cohort (Fig. 5a). Low risk score values reflected high expression levels of EpEX and Sox2, which were predictive of improved OS, DFS and DSS with hazard ratios of HR 0.44 (95% CI 0.2–0.96), 0.48 (95% CI 0.25–0.91), 0.23 (95% CI 0.05–1.13), and significant p-values (p = 0.034; p = 0.02; p = 0.048), for OS, DFS and DSS, respectively (Fig. 5a). The integrated risk score was furthermore prognostic for HPV-negative patients within the LMU cohort with respect to endpoints OS and DFS (Fig. 5a). In order to validate the risk score, we transferred the prognostic model that was developed using LMU data to data on the Göttingen cohort. Similarly, low risk score was associated with improved OS and DFS in the Göttingen cohort, thus confirming its prognostic power (Fig. 5b). However, the integrated risk factor was not prognostic for HPV-negative patients within the Göttingen cohort (Fig. 5b).

**Figure 6:**
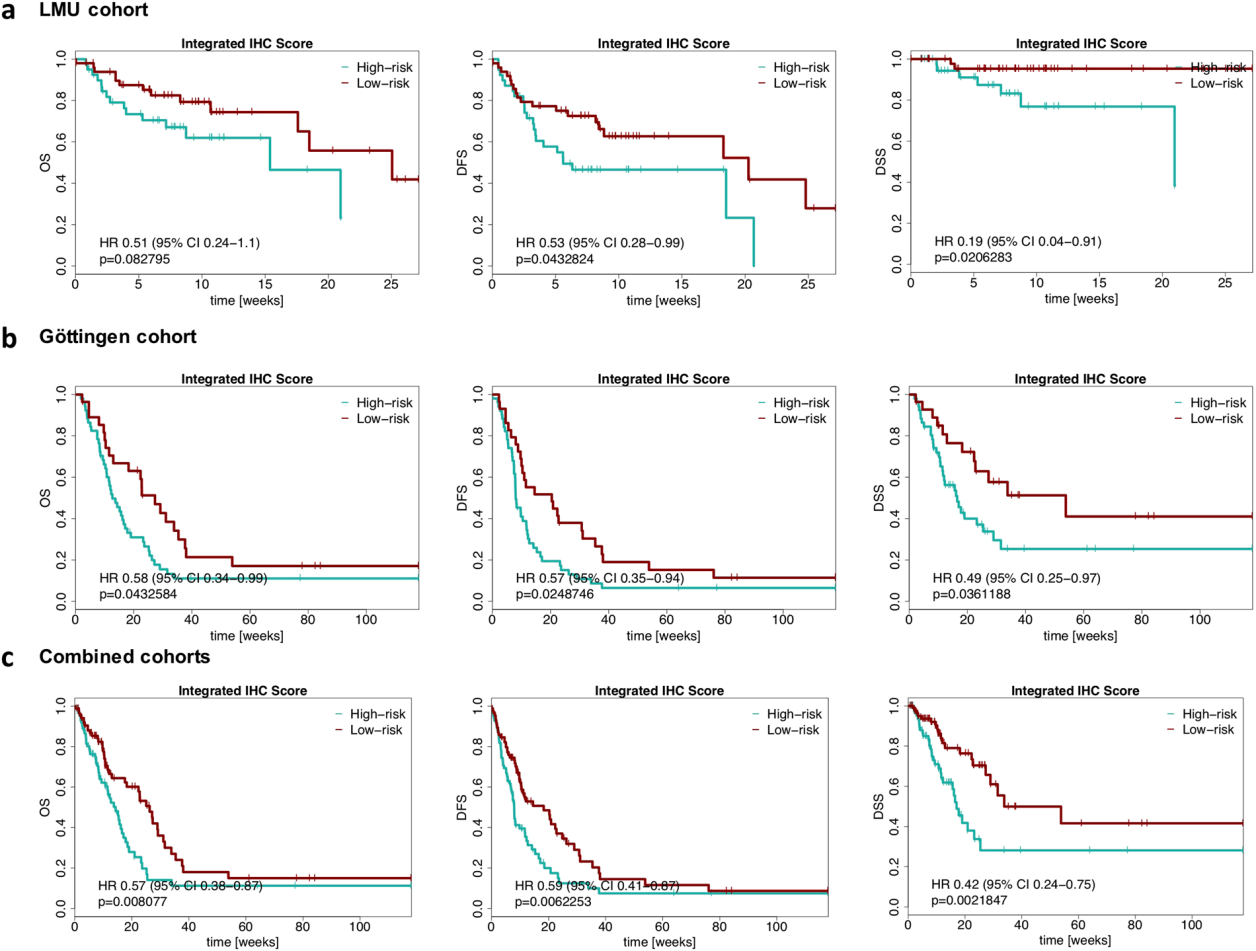
Risk factor as a prognosticator for HNSCC. (**a–c**) Optimized thresholds for Sox2 and EpEX IHC scores were implemented in an integrated risk factor to predict the clinical outcome of HSNCCs based on HPV-negative patients of the LMU cohort. Overall survival (OS), disease-free survival (DFS) and disease-specific survival (DSS) are depicted as Kaplan-Meier curves with hazard ratio (HR) at indicated 95% confidence interval (CI) and log-rank test p-values for the entire LMU cohort (**a**), Göttingen cohort (**b**), and the pooled LMU and Göttingen cohort (**c**).

In a second set of analysis, the cox model was fitted within the HPV-negative LMU cohort data followed by calculation of the risk scores and a median threshold, allowing assortment of patients into high- and low-risk in the entire LMU cohort. IHC scores of EpEX and Sox2 in relation to the risk score and Kaplan Meier plots are depicted for OS, DFS and DSS of the LMU cohort (Fig. 6a). Low risk values (*i*.*e*. high levels of EpCAM and Sox2 in the HPV-negative LMU cohort) were predictive of improved DFS and DSS (HR 0.51, 95% CI 0.28–0.99, p = 0.043; HR 0.19, 95% CI 0.04–0.91, p = 0.02,  respectively). Furthermore, low risk values had a tendency to predict improved OS, however results were not statistically significant (HR 0.51, 95% CI 0.24–1.1, p = 0.08).

The fit coefficients and the threshold determined in the HPV-negative discovery cohort (LMU cohort) were transferred to the entire validation cohort (Göttingen cohort) to calculate risk scores for subsequent assignment of patients to risk groups (Fig. 6b). Low risk values were predictive of improved OS, DFS and DSS (HR 0.58, 95% CI 0.34–0.99, p = 0.043; HR 0.57, 95% CI 0.35–0.94, p = 0.02; HR 0.49, 95% CI 0.25–0.97, p = 0.036, respectively). Lastly, both cohorts of HNSCC patients were pooled and stratified using the risk score generated in the HPV-negative LMU cohort. Low risk values were predictive of improved OS, DFS and DSS (HR 0.57, 95% CI 0.38–0.87, p = 0.008; HR 0.59, 95% CI 0.41–0.87, p = 0.0062; HR 0.42, 95% CI 0.24–0.75, p = 0.0021, respectively). Hence, risk scores based on EpCAM and Sox2 expression in HPV-negative HNSCC patients allow to stratify HNSCC patients independently of their HPV-status and treatment modality.

## Discussion

HNSCC display outstandingly high inter- and intratumoral heterogeneity^5,6^ that might account for the high rate of radio(chemo)-resistance and treatment failure. Two aspects of cellular heterogeneity are considered central to tumor progression, treatment response and, thus, clinical outcome of carcinoma patients: (*i*) The presence of cancer stem cells (CSC) within primary tumors, which are considered the source of primary tumors, recurrences and metastatic outspread^33^, (*ii*) and phenotypic changes along the EMT, which facilitate tumor cell dissemination and metastases formation, and the induction of stemness^8,9,11,12,34^. One gene locus frequently affected by mutation in HNSCC is the *SOX2* gene through amplification on chromosome 3q26^30^. Sox2 is a pluripotency factor that regulates HNSCC CSC fate through a PI3K/mTOR/Sox2/ALDH axis, which generates a tumor-initiating population with the capacity for asymmetric division and proliferation^35^. Furthermore, Sox2 fosters an epithelial phenotype in lung cancer, which has an etiology comparable to HNSCC with respect to risk factors, through enhanced transcription of the pan-carcinoma marker EpCAM^36^. In line with this concept, loss of Sox2 expression in HNSCC resulted in up-regulated vimentin expression and migration, and reduced overall and progression-free survival^30^. Induction of EpCAM results in various cellular outcome including cell-cell adhesion^37^, enhanced tumor cell proliferation^19,38^ and regulation of stem cell features^20,39–41^. Itself, EpCAM is prone to substantial regulation throughout cancer progression^27,42,43^, stem cell differentiation^22^, and during EMT related to treatment resistance, *e*.*g*. in prostate cancer^26^. Loss of EpCAM in mesodermally differentiating embryonic stem cells (ESC) during gastrulation of mouse embryos is an early event initiating at E7.0^22^. The resulting spatiotemporal patterning of EpCAM, with retention in endodermal but complete loss in mesodermal tissue, is mandatory for the completion of ESC differentiation^22^. Hence, loss of EpCAM in EMT and, generally, in mesenchymal differentiation is not only concomitant, but appears causal.

For the case of HNSCC, single cell RNA-sequencing data confirmed high inter- and intratumoral heterogeneity amongst tumor cells, but far less in associated stromal and immune cells^32^. Analysis of single cell transcriptomes allowed to extract cell signatures defining cell cycle progression, hypoxia, cell stress, and epithelial and mesenchymal differentiation statuses in HNSCC^32^. The latter two signatures were inversely correlated and characterized, amongst others, by high-level EpCAM and vimentin/slug expressions, respectively. Hence, EpCAM represents an excellent measure for the level of epithelial differentiation of carcinoma cells in head and neck tumors, whereas vimentin depicts a gradual mesenchymal switch. These molecular networks with reported functionality in tumor progression and treatment resistance prompted us to investigate protein co-expression levels and patterns of Sox2, EpCAM and vimentin in HNSCC. For all three antigens, inter- and intratumoral heterogeneity was observed, which translated into different clinical outcome. Low expression of Sox2 significantly correlated with poor OS, DFS and DSS in an univariate analysis within our discovery cohort, which is confirmatory of Bayo *et al*.^30^. Similarly, Chung *et al*. and Bochen *et al*. reported on a positive prognostic value of high-level expression of Sox2 in HNSCC^44,45^. However, contradicting results have been reported too, such that HNSCC patients with high-level Sox2 expression were characterized by poorer outcome^46^, as well as a complete lack of prognostic value of Sox2 in HNSCC^47^. Our own findings clearly are in support of a beneficial effect of high-level Sox2 expression on clinical outcome.

Low expression of epithelial marker EpCAM significantly correlated with decreased DFS and DSS (univariate). These findings contradict the reported poor prognosis of breast, colorectal, pancreatic, ovarian and bladder cancers over-expressing EpCAM^48–56^, but is in line with the association of high levels of EpCAM with improved prognosis of colonic, gastric and renal cancer^57–59^. We further confirmed our findings in a validation cohort composed of biospecimen obtained from HNSCC patients treated with primary radio(chemo)therapy. Limitations must be noted, as patients within the validation cohort were characterized by the presence of significantly larger tumors and generally more advanced disease, including increased locoregional and distant metastases (Table 2). Expectedly, these patients were confronted with substantially poorer outcome than patients included in the LMU cohort. Furthermore, EpCAM expression was assessed with two distinct monoclonal antibodies with slightly differing binding affinities (Vu1D9 > Ber-Ep4; own unpublished data), which might affect the quantification of antigen levels and explain generally lower levels of EpCAM in the Göttingen cohort. Nonetheless, EpCAM and Sox2 possessed prognostic value, even under these testing conditions and in the presence of the primary tumor throughout the observation period (Göttingen cohort). Hence, we could show that EpEX^high^ and Sox2^high^ are prognosticators of improved clinical outcome in two independent, retrospective clinical cohorts despite limitations and challenges arising from differing clinical cohorts and staining procedures. Both cohorts cover both major clinical treatment modalities and staining procedures (cryopreserved native samples versus FFPE routine staining), and should as such allow for more generalized findings.

Generally, our findings suggest that an epithelial, proliferative Sox2^high^/EpCAM^high^ phenotype of HNSCC could foster survival, which might be based on enhanced therapy response. Oppositely, loss of epithelial traits during partial EMT, as measured by EpCAM reduction and gain in vimentin^32^, might be associated with decreased proliferation and increased migration, or primarily with increased treatment resistance as demonstrated for prostate, pancreatic, breast cancer and HNSCC^15,16,26,32^. Both features can promote reduced radio(chemo)sensitivity and increased local invasion, which represent potential sources of recurrence and locoregional spread, which is a common and frequent feature of HNSCC^1^.

HPV infection is an accepted clinical factor for HNSCC^60^ that predicts improved outcome and has been implemented in the AJCC 8^th^ edition of the TNM classification of patients^4,61^. Although HPV-status as such is an important stratificator it is currently only used to distinguish two separate HNSCC entities for diagnostic/prognostic purposes, while its actual use for therapeutic stratification in routine remains under investigation in several clinical studies (RTOG 3311, NRG HN002). Furthermore, it currently leaves HPV-negative HNSCC patients without any further stratification options except for the TNM status and extracapsular extension. In this respect, EpEX^high^ qualifies as a candidate to stratify HPV-negative HNSCC into further prognostic groups. In fact, HPV-negative patients with high levels of EpCAM displayed improved survival rates for all tested clinical endpoints that were similar to that of the HPV-positive subgroup. Thus, EpEX^high^ bears the potential as a prognostic biomarker for HPV-negative HNSCC patients.

In a further step, we combined EpEX and Sox2 levels in an integrated risk factor. We deliberately did not include HPV as a parameter in the risk model owing to its impact as a strong prognostic factor in HNSCC, which might result in a risk factor that predicts HPV rather than survival. This is demonstrated by a very strong association (p < 10^−12^, not HPV-stratified LMU cohort, p < 2.2^−16^ not HPV-stratified Göttingen cohort, Fisher’s exact test) of HPV status with the risk factor generated including HPV-status and a much weaker association when not including HPV (p = 0.17, not HPV-stratified LMU cohort, 0.045 not HPV-stratified Göttingen cohort, Fisher’s exact test). In contrast, we were aiming to test whether EpCAM and Sox2 in combination are able to predict survival independent of HPV. Both EpCAM and Sox2 are only weakly associated with HPV status in the LMU cohort but not in the Göttingen cohort (p-values LMU: EpCAM 0.01, Sox2: 0.02, Göttingen: EpCAM 0.36, Sox2: 0.12). This suggests that Sox2 and EpCAM expressions are somewhat but not strongly influenced by HPV, and hence are not a strong proxy of the HPV status. Consequently, we assumed that a risk score integrating Sox2 and EpCAM but not HPV should be able to predict clinical outcome independently of HPV. In fact, the integrated risk score that combined measurements on Sox2 and EpEX expression levels significantly predicted outcome with respect to OS, DFS and DSS in both non-HPV stratified cohorts. Sox2^high^/EpEX^high^ defined patients with better outcome, both, after standard therapy involving surgery and adjuvant radio-chemotherapy, as well as after primary radio(chemo)therapy in the absence of prior surgical tumor resection. Both therapeutic strategies represent the two major treatment regimens and, thus, the risk score is applicable to the majority of clinical HNSCC cases. Additionally, Sox2 and EpCAM are common antigens for the automated staining of formalin-fixed, paraffin-embedded specimen, which would allow for comparably simple assessment in clinical routine. It must be noted that the integrated risk score was characterized by hazard ratios and p-values similar to univariate analyses of EpEX and Sox2 in both cohorts. However, although the integrated risk score does not outperform single EpEX and Sox2 scores with respect to prognostic power, implementing two or even more independent measurements within one risk score could contribute to improve the reliability of prognosis and ease handling of otherwise singular score values in clinical routine.

Additionally, in order to rule out a contribution of the HPV-status to the risk score, the cox model to determine thresholds for optimized stratification splits was fitted within the HPV-negative patients of the discovery cohort. The determined risk scores were then applied to the entire discovery and validation cohorts as well as to a pooled cohort composed of both separate cohorts. By doing so, we demonstrated that risk scores based on EpCAM and Sox2 expression generated in HPV-negative patients have the potential to predict clinical endpoints such as OS, DFS, and DSS independently of the HPV-status of the patient and of differential treatment regimens.

The integrated risk score could be assessed from pre-operative biopsies at the time point of first diagnosis and serve as an additional molecular parameter. Together with other clinical parameters such as resection margins and extracapsular extension, it could help to identify high-risk situations and, thus, the need for intensification of multimodal therapy.

In summary, the integrated risk factor, and EpEX^high^ more specifically, presented herein have the potential to contribute to improve HNSCC stratification of HNSCC patients, including HPV-negative cases, and might open novel options for therapy decisions.

## Methods

### Human biospecimen

Biospecimen are reported according to BRISQ and REMARK standards^62^. The Ludwig-Maximilians-University of Munich (LMU) cohort (discovery cohort) is composed of 94 patients with HNSCC of whom tumor biomaterial has been collected. The cohort comprises primary tumors (n = 81), recurrences (n = 3), residual carcinoma (n = 1) and secondary carcinomas of the head and neck (n = 9). Furthermore, distant normal mucosa and tumor-adjacent normal mucosa were available for 87 and 19 of these patients, respectively. Patients were treated with surgery and adjuvant radio(chemo)therapy upon indication. Age, sex, tumor localization, smoking habits, TNM and p16 status, as a surrogate marker for HPV infection^63,64^, are compiled in Table 1. P16 status will be referred to as HPV-status in the following. The most prevalent HNSCC subgroup within the cohort comprised oropharyngeal squamous carcinomas (57.4%), with 30.9% HPV-positive samples, thus conforming to overall frequencies worldwide^65^. All patients of the LMU cohort received surgical treatment and adjuvant radiotherapy (radiation doses: 50.4–70 Gy; mean 63.07 Gy; median 64.0 Gy; three patients obtained increased doses up to 70 Gy based on clinical requirements such as uncertain resection margins with residual tumor *in situ*) with or without simultaneous chemotherapy. Most patients received cisplatin/5-fluorouracil (CDDP/5-FU). In selected cases, mitomycin C (MMC), 5-FU/MMC, or Cetuximab replaced platin-based chemotherapy. Macroscopically normal mucosa was obtained after surgical removal of the primary tumor beyond the resection margins with >5 mm distance to the initial tumor bed. Tumor-adjacent mucosa was defined as areas of histologically normally structured but hyper- to dysplastic epithelium in the close vicinity of tumor fields. All biospecimens were stabilized through embedment in tissue-Tek® (Sakura, Finetek, The Netherlands), snap-frozen in liquid nitrogen, and preserved at −80 °C before further processing.

The Göttingen cohort (validation cohort) is composed of 94 patients with HNSCC, who received definitive radiotherapy with or without simultaneous chemotherapy. The cohort comprises 94 primary tumors. Age, sex, tumor localization, smoking habits, TNM and p16 status are compiled in Table 1. Intended total radiation dose in this primary setting was 70.0 and 66.0 for 87 and 7 patients, respectively. Complete radiation as planned was administered in 85 individuals (90.4%); total dose was reduced in six patients by <10.0 and in three by 10.0–20.0 Gy due to medical reasons or according to patient willingness. Concomitant chemotherapy was given in 75 out of the 94 patients of whom 41 received 5-FU/MMC, 30 cisplatin as single agent, and four received Cetuximab. Biospecimens of the Göttingen cohort were formalin-fixed and paraffin-embedded before processing as tissue microarrays.

### Immunohistochemistry and immunofluorescence staining

Immunohistochemistry intensity scores (IHC score) were calculated as the product of intensity (0 to 3 +) and percentage of expressing tumor cells within biospecimens. IHC scores represent averages of values independently assessed by minimum two experimenters, who were blinded with respect to clinical staging and outcome of patients. Staining was analyzed separately for each individual antigen in order to preclude potential bias with respect to correlation of antigen expression. Antigen expression in the LMU cohort was assessed in 2–4 sections (≥10 × 10 mm) of primary specimens and represented average IHC scores across all entire sections considering tumor areas only. Antigen expression in the Göttingen cohort was assessed in tissue microarrays of biopsies from primary tumor specimens and represented the average of 2–4 punches of 1.5 mm diameter taken randomly from different regions within each specimen. The comparably large size of sample within the LMU cohort and the number of punches and diversity of areas for samples within the Göttingen cohort were chosen to allow for the most adequate coverage of tumor heterogeneity.

EpEX- (LMU cohort: VU1D9, Cell Signaling Technology, NEB, Frankfurt, Germany, #2929; Göttingen cohort: Ber-Ep4, Dako, Hamburg, Germany, #M080429), Sox2- (D6D9, Cell Signaling Technology, NEB, Frankfurt, Germany, #3579), vimentin- (3B4, Dako, Hamburg, Germany, #M702001), CD31- (JC70A, Dako, Hamburg, Germany, #IR61061), CD68- (KP1, Dako, Hamburg, Germany, #M081401), and CD90-specific antibodies (2Q1469, DCS Immuno Line, Hamburg, Germany, #CI921C002) were used for immunohistochemistry and immunofluorescence detection of antigens. Immunostaining was performed using the avidin-biotin-peroxidase method (Vectastain, Vector laboratories, Burlingame, CA, USA) according to the manufacturers’ protocol. For immunofluorescence, Alexa Fluor®−488- and Alexa Fluor®−594-conjugated secondary antibodies were used to visualize specific primary antibodies. Laser scanning confocal microscopy images were recorded with a TCS-SP5 system (Leica Microsystems; Wetzlar, Germany).

### Clinical endpoints and survival analysis

Overall survival (OS), disease-free survival (DFS) and disease-specific survival (DSS) were chosen as clinical endpoints. We calculated OS (months) from the date of diagnosis to death due to any cause, DFS to the first observation of any recurrence or death, and DSS to the date of HNSCC-related death. In the absence of an event, patients were censored at the date of the last follow-up visit.

Data analysis was performed in R (R Core Team, R: A Language and Environment for Statistical Computing, R Foundation for Statistical Computing, 2017; version 3.4.0) in combination with R-survival package (CRAN). For univariate analysis, the IHC expression scores were included into cox-proportional hazard models after binarization into high- and low expressors. The threshold for binarization was the value picked from the whole range of expression scores that resulted in a maximum split of high- and low-expressors with regard to hazard-ratio. In order to prevent artificially extreme hazard-ratios resulting from very small groups only thresholds were allowed that split patients in groups containing at least 5 patients. Hazard ratios, 95% confidence interval ratios, median survival times and log-rank p-values were calculated and included in Kaplan-Meier plots. In order to generate prognostic models integrating EpEX and Sox2, in a first step a multivariate model was built with the continuous expression values of the two proteins included as covariates. The resulting fit coefficients were extracted from the model and multiplied with the appropriate expression values of each patient before building the sum, which was then used as risk score. The risk scores were binarised into high- and low-risk by a threshold that was maximized for hazard-ratio as described above. Hazard ratios, 95% confidence interval ratios, median survival times and log-rank p-values were calculated and included in Kaplan-Meier plots along with distribution plots of risk scores and expression heatmaps. Statistical tests for all figures are justified as appropriate. Visual inspection of distribution shapes of comparison groups revealed no obvious differences. Normality was tested with the Kolgomorov-Smirnov test and assumptions for the test were met. Equal variances between the groups were tested using the Levene test with no differences observed.

### Analysis of the TCGA HNSCC cohort

The results are based upon data generated by the TCGA Research Network: http://cancergenome.nih.gov. mRNA expression level z-scores for EpCAM, Sox2 and Vimentin were downloaded for the Nature published HNSCC TCGA cohort (n = 279) at cBioPortal^5,66,67^. Spearman correlation analyses of mRNA expression z-scores were performed, and results were considered statistically significant when p < 0.05.

### Ethics approval and consent to participate

All clinical samples were obtained after written informed consent during routine surgery or biopsy based on the approval by the ethics committee of the local medical faculties (Ethikkomission der Medizinischen Fakultät der Ludwig-Maximilians-Universität; #087-03; #197-11; #426-11; Ethikkomission der Medizinischen Fakultät der Universität Göttingen; #7/4/2012) and in compliance with the WMA Declaration of Helsinki and the Department of Health and Human Services Belmont Report.

## Electronic supplementary material

Supplementary Figures

## Data Availability

Data availability is restricted as required by the ethics obligations and relevant medical and legal issues.
