# Supplementary material for: High Expression of EpCAM and Sox2 is a Positive Prognosticator of Clinical Outcome for Head and Neck Carcinoma
Source: Sci Rep. 2018 Oct 1;8:14582. doi: 10.1038/s41598-018-32178-8 (PMC6167386; doi:10.1038/s41598-018-32178-8)
Supplement: Supplementary file 1 — Supplementary Figures [file 41598_2018_32178_MOESM1_ESM.pdf]

High Expression of EpCAM and Sox2 is a Positive Prognosticator of Clinical Outcome for Head and Neck Carcinoma

Philipp Baumeister [1,2], Alessandra Hollmann [1*], Julia Kitz [3*], Artemis Afthonidou [1], Florian Simon [1], Julius Shakhtour [1], Brigitte Mack [1], Gisela Kranz [1], Darko Libl [1], Martin Leu [4], Markus A. Schirmer [4], Martin Canis [1], Claus Belka [2,5], Horst Zitzelsberger [2,6], Ute Ganswindt [2,5], Julia Hess [2,6], Mark Jakob [1,§], Kristian Unger [2,6,§], and Olivier Gires [1,2,§]*

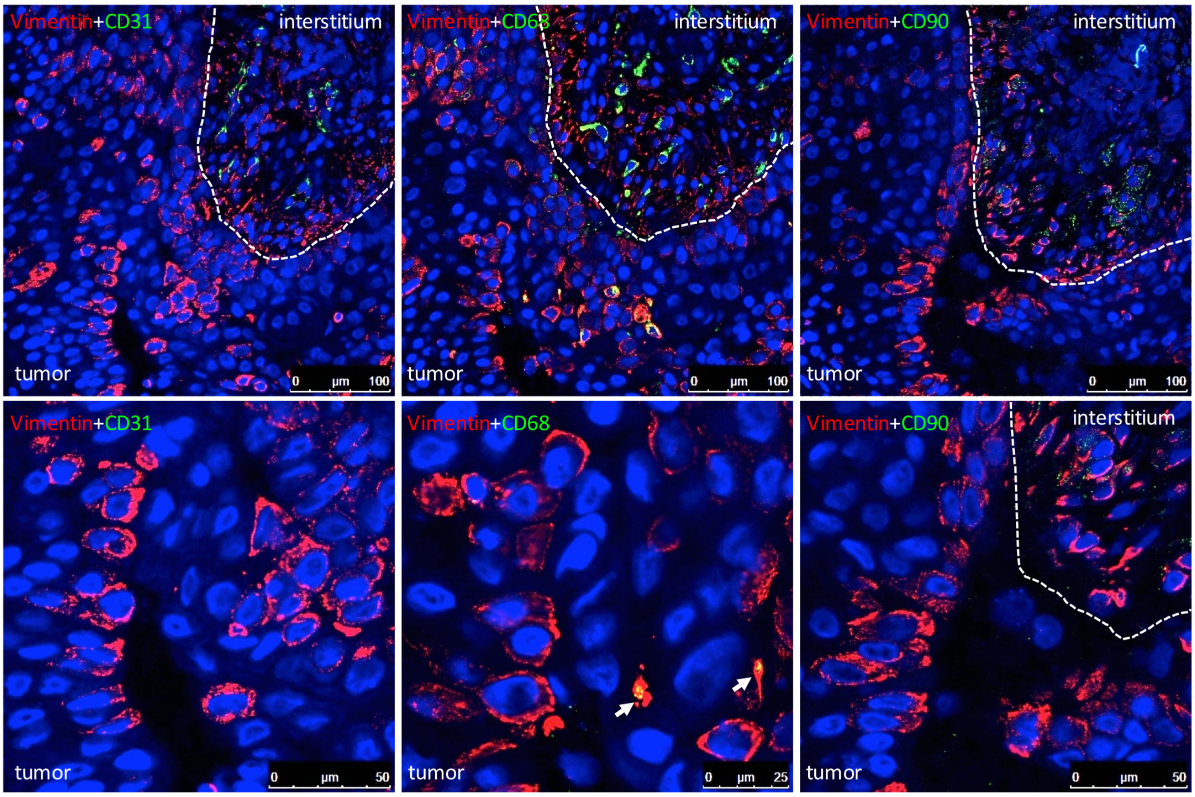

Supplementary Figure 1 - Detection of CD31, CD68 and CD90 in HNSCC.

CD31 (endothelial cells, monocytes, neutrophils), CD68 (monocytes, macrophages) and CD90 (fibroblasts, stem cells, thymocytes) expression was analyzed in simultaneous immunofluorescence staining with vimentin one HNSCC specimen. All markers were depicted as red staining except for vimentin (green). Nuclear DNA is stained in blue with DAPI. The margin between tumor and interstitium are marked as doted lines. Vimentin^{+}/CD68^{+} cells are marked with white arrows. Lower panels show higher magnification of selected areas.

Supplementary Table 1: IHC scores of EpEX, Sox2, and vimentin expression in HNSCC primary tumors of the LMU cohort (n=94). Scores have been evaluated by 3 (EpEX and Sox2) and 2 (vimentin) experimenters and are shown as single and mean values. Data on gender, age, smoking behavior, p16 expression as a surrogate for HPV status, HNSCC tumor localization as well as T and N status for the study participants of the LMU cohort are listed beneath.

Patient	EpEx IHC score 1	EpEx % x int.	EpEx IHC score 2	EpEx % x int.	EpEX IHC score 3	EpEx % x int.	Mean EpEx IHC score
**1**	300	100*3	270	90*3 10*0	300	100*3	290.0
**2**	130	20*3 20*2 30*1 30*0	240	70*3 10*2 10*1 10*0	150	30*3 20*2 20*1 30*0	173.3
**3**	0	100*0	0	100*0	0	100*0	0.0
**4**	180	10*3 70*2 10*1 10*0	190	10*3 80*2 10*0	120	40*2 40*1 20*0	163.3
**5**	280	90*3 10*1	280	80*3 20*2	250	50*3 50*2	270.0
**6**	290	90*3 10*2	150	50*2 50*1	300	100*3	246.7
**7**	280	90*3 5*2 5*0	300	100*3	300	100*3	293.3
**8**	280	80*3 20*2	250	70*3 20*2 10*0	280	80*3 20*2	270.0
**9**	170	20*3 30*2 50*1			230	50*3 30*2 20*1	200.0
**10**	110	30*3 10*2 60*0	140	40*3 10*2 50*0	150	40*3 10*2 10*1 40*0	133.3
**11**	180	20*3 40*2 40*1			180	20*3 50*2 20*1 10*0	180.0
**12**	190	20*3 60*2 10*1 10*0	110	40*2 30*1 30*0	220	50*3 20*2 30*1	173.3
**13**	290	90*3 10*2			270	70*3 30*2	280.0
**14**	230	60*3 10*2 30*1	240	80*3 20*0	240	80*3 20*0	236.7
**15**	230	50*3 30*2 20*1			210	50*3 20*2 20*1 10*0	220..0
**16**	170	30*3 20*2 40*1 10*0			230	50*3 30*2 20*1	200.0
**17**	240	60*3 20*2 20*1	220	70*3 10*1 20*0	250	80*3 10*1 10*0	236.7
**18**	160	20*3 50*2 30*0	170	70*2 30*1	115	10*3 30*2 25*1 35*0	148.3
**19**	270	80*3 10*2 10*1	280	80*3 20*2	280	90*3 10*1	276.7
**20**	180	30*3 30*2 30*1 10*0			210	30*3 50*2 20*1	195.0
**21**	230	70*3 20*1 10*0	280	80*3 20*2	220	60*3 20*2 20*0	243.3
**22**	160	20*3 30*2 40*1 10*0			210	50*3 20*2 20*1 10*0	185.0
**23**	150	20*3 30*2 30*1 20*0	230	30*3 70*2	250	50*3 50*2	210.0
**24**	150	50*3 50*0			170	40*3 20*2 10*1 30*0	160.0
**25**	260	70*3 20*2 10*1			290	90*3 10*2	275.0
**26**	190	40*3 30*2 10*1 10*0	260	80*3 20*1	280	80*3 20*2	243.3
**27**	230	70*3 20*1 10*0	200	100*2	250	50*3 50*2	226.7

**28**	280	90*3 10*1	300	100*3	300	100*3	293.3
**29**	260	70*3 20*2 10*1	290	90*3 10*2	300	100*3	283.3
**30**	110	20*2 70*1 10*0	100	100*1	130	40*2 50*1 10*0	113.3
**31**	140	30*3 20*2 10*1 60*0	180	40*3 30*2 30*0	190	50*3 20*2 30*0	170.0
**32**	280	80*3 20*2			230	60*3 15*2* 20*1 5*0	255.0
**33**	15	5*3 95*0	70	30*2 10*1 60*0	80	30*2 20*1 50*0	55.0
**34**	220	60*3 10*2 20*1 10*0	230	60*3 20*2 10*1 10*0	250	60*3 30*2 10*0	233.3
**35**	70	20*3 10*1 70*0			280	80*3 20*2	175.0
**36**	290	90*3 10*2	280	80*3 20*2	300	100*3	290.0
**37**	6	2*3 98*0			0	100*0	3.0
**38**	265	80*3 10*2 5*1 5*0	280	80*3 20*2	290	90*3 10*2	278.3
**39**	15	5*3 95*0			35	5*3 10*2 85*0	25.0
**40**	270	70*3 30*2			200	30*3 40*2 30*1	235.0
**41**	230	70*3 20*1 10*0	250	80*3 10*1 10*0	220	60*3 10*2 20*1 10*0	233.3
**42**	0	100*0			5	5*1 95*0	2.5
**43**	170	20*3 50*2 10*1 20*0	255	70*3 20*2 5*1 5*0	230	60*3 20*2 10*1 10*0	218.3
**44**	40	5*3 25*1 70*0	20	20*1 80*0	70	5*3 20*2 15*1 60*0	43.3
**45**	260	70*3 20*2 10*1			290	90*3 10*2	275.0
**46**	110	30*3 10*2 60*0	150	30*3 30*2 40*0	140	40*3 20*1 40*0	133.3
**47**	130	40*3 10*1 50*0			220	70*3 10*1 20*0	175.0
**48**	170	30*3 30*2 20*1 20*0	240	70*3 10*2 10*1 10*0	260	60*3 40*2	223.3
**49**	70	10*3 10*2 20*1 60*0	140	30*3 20*2 10*1 40*0	120	50*2 20*1 30*0	110.0
**50**	300	100*3	300	100*3	300	100*3	300.0
**51**							
**52**	80	10*2 60*1 30*0	40	20*2 80*0	80	10*2 60*1 30*0	66.7
**53**	280	90*3 10*1	300	100*3	300	100*3	293.3
**54**	300	100*3	210	10*3 90*2	300	100*3	270.0
**55**	240	70*3 10*2 10*1 10*0			260	80*3 20*1	250.0
**56**	210	50*3 30*2 20*0	290	90*3 10*2	270	70*3 30*2	256.7
**57**	180	30*3 30*2 30*1 10*0			210	30*3 50*2 20*1	195.0
**58**	160	30*3 20*2 30*1 20*0	250	70*3 20*2 10*0	180	40*3 20*2 20*1 20*0	196.7
**59**	200	30*3 40*2 30*1	190	10*3 80*2 10*0	260	70*3 20*2 10*1	216.7
**60**	60	10*3 30*1	30	5*2 20*1	80	30*2 20*1	56.7

		60*0		75*0		50*0	
**61**	185	20*3 50*2<br>25*1 5*0	170	10*3 70*2<br>20*0	250	60*3 30*2<br>10*1	201.7
**62**	300	100*3	270	90*3 10*0	280	80*3 20*2	283.3
**63**	190	40*3 30*2<br>10*1 20*0	150	50*3 50*0	250	80*3 10*1<br>10*0	196.7
**64**	170	30*3 20*2<br>40*1 10*0	110	30*3 10*2<br>60*0	160	40*3 20*2<br>40*0	146.7
**65**	260	60*3 40*2			220	30*3 65*2<br>5*0	240.0
**66**	200	30*3 40*2<br>30*1	250	50*3 50*2	200	30*3 40*2<br>30*1	216.7
**67**	300	100*3	300	100*3	300	100*3	300.0
**68**	300	100*3	300	100*3	300	100*3	300.0
**69**	5	5*1 95*0	30	30*1 70*0	30	10*2 10*1<br>80*0	21.7
**70**	5	5*1 95*0	0	100*0	0	100*0	1.7
**71**	260	70*3 20*2<br>10*1	280	80*3 20*2	280	80*3 20*2	273.3
**72**	150	10*3 40*2<br>40*1 10*0			160	10*3 60*2<br>10*1 10*0	155.0
**73**	275	90*3 5*1<br>5*0	270	80*3 10*2<br>10*1	280	90*3 10*1	275.0
**74**	0	100*0	15	5*3 95*0	20	20*1 80*0	11.7
**75**	290	90*3 10*2			290	90*3 10*2	290.0
**76**	250	80*3 10*1<br>10*0			240	70*3 10*2<br>10*1 10*0	245.0
**77**	260	80*3 20*1	270	80*3 10*2<br>10*1	300	100*3	276.7
**78**	270	70*3 30*2	250	50*3 50*2	270	70*3 30*2	263.3
**79**	35	5*3 10*2<br>85*0	55	10*3 10*2<br>5*1 75*0	100	10*3 30*2<br>10*1 50*0	63.3
**80**	200	40*3 30*2<br>20*1 10*0	210	40*3 40*2<br>10*1 10*0	200	40*3 30*2<br>20*1 10*0	203.3
**81**	220	60*3 10*2<br>20*1 20*0	250	50*3 50*2	280	80*3 20*2	250.0
**82**	160	10*3 40*2<br>50*1			180	30*3 40*2<br>10*1 20*0	170.0
**83**	80	10*3 25*2<br>65*0			175	30*3 30*2<br>25*1 15*0	127.5
**84**	270	70*3 30*2			250	60*3 30*2<br>10*1	260.0
**85**	285	90*3 5*2<br>5*1	285	95*3 5*0	300	100*3	290.0
**86**	280	90*3 10*1	280	80*3 20*2	300	100*3	286.7
**87**	235	60*3 20*2<br>15*1 5*0	120	60*2 40*0	245	70*3 10*2<br>15*1 5*0	200.0
**88**	200	50*3 20*2<br>10*1 20*0	280	80*3 20*2	210	50*3 20*2<br>20*1 10*0	230.0
**89**	100	10*3 10*2<br>50*1 30*0	50	50*1 50*0	90	10*3 20*2<br>20*1 50*0	80.0
**90**	170	40*3 10*2<br>30*1 20*0	290	90*3 10*2	230	50*3 30*2<br>20*1	230.0
**91**	150	20*3 30*2<br>30*1 20*0			190	20*3 50*2<br>30*1	170.0
**92**	85	5*3 5*2<br>60*1 30*0	90	10*3 60*1<br>30*0	160	20*3 40*2<br>20*1 20*0	111.7

**93**	270	70*3 30*2	270	70*3 30*2	280	80*3 20*2	273.3
**94**	290	90*3 10*2	300	100*3	300	100*3	296.7

**Patient**	**Sox2 IHC score 1**	**Sox2 % x int.**	**Sox2 IHC score 2**	**Sox2 % x int.**	**Sox2 IHC score 3**	**Sox2 % x int.**	**Mean Sox2 IHC score**
**1**	0	100*0	0	100*0	0	100*0	0.0
**2**	120	20*3 20*2 20*1 40*0	240	70*3 10*2 10*1 10*0	140	30*3 20*2 10*1 40*0	166.7
**3**	0	100*0	10	10*1 90*0	90	40*2 10*1 50*0	33.3
**4**	0	100*0	0	100*0	0	100*0	0.0
**5**	30	30*1 70*0	5	5*1 95*0	20	20*1 80*0	18.3
**6**	240	80*3 20*0	150	50*2 50*1	70	10*3 20*2 70*0	153.3
**7**	185	10*3 70*2 15*1 5*0	100	20*3 20*2 60*0	170	40*3 20*2 10*1 30*0	151.7
**8**	10	10*1 90*0	10	10*1 90*0	30	10*2 10*1 80*0	16.7
**9**	0	100*0			0	100*0	0.0
**10**	170	30*3 40*2 30*0	140	40*3 10*2 50*0	210	60*3 10*2 10*1 20*0	173.3
**11**	10	10*1 90*0			10	10*1 90*0	10.0
**12**	40	10*2 20*1 70*0	90	30*2 30*1 40*0	10	10*1 90*0	46.7
**13**	220	50*3 30*2 10*1 10*0			220	70*3 10*1 20*0	220.0
**14**	200	30*3 40*2 30*1	180	20*3 60*2 20*0	120	50*2 20*1 30*0	166.7
**15**	90	20*3 10*2 10*1 60*0			150	40*3 10*2 10*1 40*0	120.0
**16**	140	30*3 20*2 10*1 40*0			100	20*3 20*2 60*0	120.0
**17**	190	30*3 40*2 20*1 10*0	120	30*3 30*1 40*0	200	50*3 20*2 10*1 20*0	170.0
**18**	60	30*2 70*0	55	5*3 40*1 55*0	120	20*3 20*2 20*1 40*0	78.3
**19**	230	60*3 20*2 10*1 10*0	270	70*3 30*2	280	90*3 10*1	260.0
**20**	130	10*3 30*2 40*1 20*0			170	40*3 20*2 10*1 30*0	150.0
**21**	240	70*3 10*2 10*1 10*0	280	80*3 20*2	200	60*3 10*2 30*0	240.0
**22**	70	10*3 10*2 20*1 60*0			130	10*3 40*2 20*1 30*0	100.0
**23**	0	100*0	0	100*0	5	5*1 95*0	1.7
**24**	230	70*3 10*2 20*0			220	60*3 20*2 20*0	225.0
**25**	240	60*3 20*2 20*1			255	70*3 20*2 5*1 5*0	247.5
**26**	230	50*3 30*2 20*1	160	80*2 20*0	180	40*3 20*2 20*1 20*0	190.0
**27**	0	100*0	100	100*1	0	100*0	33.3
**28**	120	40*2 40*1 20*0	100	40*2 20*1 40*0	190	50*3 20*2 30*0	136.7

**29**	35	5*3 20*1 75*0	60	20*3 80*0	50	10*3 10*2 80*0	48.3
**30**	80	80*1 20*0	40	40*1 60*0	10	10*1 90*0	43.3
**31**	170	30*3 30*2 20*1 20*0	180	40*3 30*2 30*0	200	60*3 10*2 30*0	183.3
**32**	240	60*3 20*2 20*1			220	60*3 10*2 20*1 10*0	230.0
**33**	0	100*0	30	30*1 70*0	0	100*0	10.0
**34**	30	30*1 70*0	40	20*2 80*0	50	10*3 10*2 80*0	40.0
**35**	120	20*3 20*2 20*1 40*0			140	60*2 20*1 20*0	130.0
**36**	290	90*3 10*2	280	80*3 20*2	230	60*3 20*2 10*1 10*0	266.7
**37**	0	100*0			0	100*0	0.0
**38**	255	70*3 20*2 5*1 5*0	270	70*3 30*2	270	80*3 10*2 10*1	265.0
**39**	90	10*3 20*2 20*1 50*0			40	40*1 60*0	65.0
**40**	0	100*0			0	100*0	0.0
**41**	190	30*3 50*2 20*0	230	70*3 10*2 20*0	200	50*3 20*2 10*1 20*0	206.7
**42**	0	100*0			0	100*0	0.0
**43**	190	50*3 20*2 30*0	210	40*3 40*2 10*1 10*0	240	70*3 10*2 10*1 10*0	213.3
**44**	0	100*0	5	5*1 95*0	0	100*0	1.7
**45**	160	40*3 10*2 20*1 30*0			230	60*3 20*2 10*1 10*0	195.0
**46**	140	30*3 20*2 10*1 40*0	210	70*3 30*0	170	50*3 20*1 30*0	173.3
**47**	140	30*3 20*2 10*1 40*0			200	50*3 20*2 10*1 20*0	170.0
**48**	110	10*3 20*2 40*1 30*0	70	10*2 50*1 40*0	120	20*3 20*2 20*1 20*0	100.0
**49**	205	30*3 50*2 15*1 5*0	280	80*3 20*2	230	40*3 50*2 10*1	238.3
**50**	280	80*3 20*2	300	100*3	260	70*3 20*2 10*1	280.0
**51**	190	40*3 30*2 10*2 20*0					190.0
**52**	30	30*1 70*0	0	100*0	0	100*0	10.0
**53**	260	80*3 20*1	300	100*3	210	50*3 20*2 20*1 10*0	256.7
**54**	0	100*0	0	100*0	10	10*1 90*0	3.3
**55**	30	30*1 70*0			5	5*1 95*0	17.5
**56**	220	50*3 20*2 30*1	290	90*3 10*2	260	70*3 20*2 10*1	256.7
**57**	160	30*3 20*2 30*1 20*0			240	60*3 30*2 10*0	200.0
**58**	140	30*3 10*2 30*1 30*0	160	70*2 20*1 10*0	170	40*3 20*2 10*1 30*0	156.7
**59**	230	60*3 10*2 30*1	250	80*3 10*1 10*0	250	70*3 15*2 10*1 5*0	243.3
**60**	20	20*1 80*0	0	100*0	10	10*1 90*0	10.0
**61**	190	30*3 40*2 20*1 10*0	130	10*3 50*2 40*0	160	30*3 20*2 30*1 20*0	160.0
**62**	275	90*3 5*1 5*0	250	80*3 10*1	200	30*3 40*2	241.7

				10*0		30*1	
**63**	140	15*3 30*2 35*1 20*0	140	40*3 10*2 50*0	170	50*3 20*1 30*0	150.0
**64**	100	10*3 20*2 30*1 40*0	90	30*3 70*0	70	10*3 20*2 70*0	86.7
**65**	230	50*3 30*2 20*1			235	60*3 20*2 15*1 5*0	232.5
**66**	210	40*3 30*2 30*1	250	50*3 50*2	230	50*3 30*2 20*1	230.0
**67**	30	30*1 70*0	0	100*0	0	100*0	10.0
**68**	0	100*0	0	100*0	0	100*0	0.0
**69**	30	30*1 70*0	0	100*0	20	20*1 80*0	16.7
**70**	0	100*0			0	100*0	0.0
**71**	260	70*3 20*2 10*1	280	80*3 20*2	280	80*3 20*2	273.3
**72**	250	70*3 20*2 10*0			250	80*3 10*1 10*0	250.0
**73**	275	90*3 5*1 5*0	260	70*3 20*2 10*1	300	100*3	278.3
**74**	0	100*0	0	100*0	5	5*1 95*0	1.7
**75**	170	20*3 40*2 30*1 10*0			200	60*3 20*1 20*0	185.0
**76**	180	40*3 20*2 20*1 20*0			220	70*3 10*1 20*0	200.0
**77**	140	30*3 50*1 20*0	120	30*3 30*1 40*0	80	30*2 20*1 50*0	113.3
**78**	250	60*3 30*2 10*1	270	70*3 30*2	140	60*2 20*1 20*0	220.0
**79**	100	20*3 10*2 20*1 50*0	110	30*3 10*2 60*0	170	40*3 20*2 10*1 30*0	126.7
**80**	230	40*3 50*2 10*1	220	40*3 40*2 20*1	230	50*3 30*2 20*1	226.7
**81**	130	20*3 20*2 30*1 20*0	170	30*3 30*2 20*1 20*0	200	40*3 30*2 20*1 10*0	166.7
**82**	170	30*3 20*2 40*1 10*0			140	60*2 20*1 20*0	155.0
**83**	33	1*3 15*2 84*0			50	10*3 10*2 70*0	41.5
**84**	45	10*2 25*1 65*0			30	10*2 10*1 80*0	37.5
**85**	190	40*3 30*2 10*1 20*0	250	70*3 20*2 10*0	280	80*3 20*2	240.0
**86**	170	40*3 50*1 10*0	130	30*3 20*2 50*0	130	30*3 20*2 50*0	143.3
**87**	40	5*2 30*1 65*0	10	5*2 95*0	30	10*2 10*1 80*0	26.7
**88**	220	60*3 20*2 20*0	280	80*3 20*2	210	60*3 10*2 10*1 20*0	236.7
**89**	60	5*3 10*2 25*1 60*0	45	15*2 15*1 70*0	90	10*3 20*2 20*1 50*0	65.0
**90**	160	40*3 10*2 20*1 30*0	210	70*3 30*0	200	50*3 20*2 10*1 20*0	190.0
**91**	40	10*2 20*1 70*0			30	10*2 10*1 80*0	35.0
**92**	80	5*2 70*1 25*0	80	10*3 50*1 40*0	180	40*3 20*2 20*1 20*0	113.3
**93**	150	10*3 50*2	90	10*2 70*1	30	30*1 70*0	90.0

		20*1 20*0		20*0			
94	270	80*3 10*2 10*1	290	90*3 10*2	300	100*3	286.7

Patient	Vimentin IHC score 1	Vimentin % x int.	Vimentin IHC score 2	Vimentin % x int.	Mean vimentin IHC score
1	0	100*0	0	100*0	0
2	0	100*0	0	100x*0	0
3	0	100*0	90	30*3 70*0	45
4	0	100*0	20	20*1 80*0	10
5	280	80*3 20*2	300	100*3	290
6	0	100*0	100	50*2 50*0	50
7	0	100*0	0	100*0	0
8	60	60*1 40*0	60	30*2 70*0	60
9	100	100*1	260	80*3 20*1	180
10	20	10*2 90*0	0	100*0	10
11			250	50*3 50*2	125
12	0	100*0	0	100*0	0
13	75	25*3 75*0	0	100*0	37.5
14	90	10*3 10*2 40*1 40*0	20	20*1 80*0	55
15	0	100*0	0	100*0	0
16	0	100*0	0	100*0	0
17	0	100*0	0	100*0	0
18	0	100*0	0	100*0	0
19	35	5*3 20*1 75*0	0	100*0	17.5
20	0	100*0	10	10*2 90*0	5
21	0	100*0	0	100*0	0
22	20	10*2 90*0	30	30*1 70*0	25
23	160	70*2 20*1 10*0	60	60*1 40*0	110
24	0	100*0	0	100*0	0
25	0	100*0	0	100*0	0
26	0	100*0	0	100*0	0
27	90	90*1 10*0	100	100*1	95
28	30	10*3 90*0	0	100*0	15
29	170	80*2 10*1 10*0	90	90*1 10*0	130
30	0	100*0	0	100*0	0
31	30	10*3 90*0	20	20*1 80*0	25
32	30	10*3 90*0	0	100*0	15
33	190	90*2 10*1	300	100*3	245
34	110	30*3 20*1 50*0	120	40*3 60*0	115
35	200	40*3 30*2 20*1 10*0	200	100*2	200
36	100	30*2 40*1 20*0	200	100*2	150
37	200	100*2	200	100*2	200
38	0	100*0	0	100*0	0
39			0	100*0	0
40	0	100*0	40	20*2 80*0	20
41	0	100*0	0	100*0	0
42	0	100*0	0	100*0	0
43	30	10*2 10*1 80*0	120	60*2 40*0	75
44	60	60*1 40*0	190	90*2 10*1	125

**45**	0	100*0	0	100*0	0
**46**	20	10*2 90*0	20	20*2 80*0	20
**47**	0	100*0	0	100*0	0
**48**	0	100*0	0	100*0	0
**49**	20	5*2 10*1 85*0	0	100*0	10
**50**	0	100*0	0	100*0	0
**51**					140
**52**	200	100*2	300	100*3	250
**53**	200	100*2	200	100*2	200
**54**			0	100*0	0
**55**	0	100*0	20	20*1 80*0	10
**56**	15	5*3 95*0	0	100*0	7.5
**57**	15	15*1 85*0	90	90*1 100*0	52.5
**58**	0	100*0	0	100*0	0
**59**	85	5*3 30*2 10*1 65*0	90	30*3 70*0	87.5
**60**	30	10*2 10*1 80*0	80	40*2 60*0	55
**61**			0	100*0	0
**62**	20	5*3 5*1 90*0	20	10*2 90*0	20
**63**	0	100*0	0	100*0	0
**64**	0	100*0	0	100*0	0
**65**	30	10*3 90*0	0	100*0	15
**66**	10	10*1 90*0	0	100*0	5
**67**	240	40*3 60*2	120	60*2 40*0	180
**68**	0	100*0	0	100*0	0
**69**	10	10*1 90*0	20	20*1 80*0	15
**70**			0	100*0	0
**71**			0	100*0	0
**72**	0	100*0	0	100*0	0
**73**	0	100*0	0	100*0	0
**74**	0	100*0	0	100*0	0
**75**	90	30*3 70*0	120	40*3 60*0	105
**76**	30	10*3 90*0	90	30*3 70*0	60
**77**	190	90*2 10*1	300	100*3	245
**78**	50	50*1 50*0	80	80*1 20*0	65
**79**	0	100*0	40	20*2 80*0	20
**80**	0	100*0	0	100*0	0
**81**	0	100*0	0	100*0	0
**82**	0	100*0	0	100*0	0
**83**	0	100*0	0	100*0	0
**84**	30	10*3 90*0	90	30*3 70*0	60
**85**	0	100*0	0	100*0	0
**86**	15	5*3 95*0	40	20*2 80*0	27.5
**87**	30	10*3 90*0	100	100*1	65
**88**	15	5*3 95*0	0	100*0	7.5
**89**	0	100*0	0	100*0	0
**90**	30	10*3 90*0	45	15*3 85*0	37.5
**91**	160	20*3 50*2 30*0	270	90*3 10*0	215
**92**	0	100*0	0	100*0	0
**93**	15	5*3 95*0	0	100*0	7.5
**94**	80	10*3 50*1 40*0	20	20*1 80*0	50

Patient	Gender	Age	Age groups	Smoking status	p16	Localisation
**1**	male	58	50-69	current	negative	Oropharynx
**2**	male	63	50-69	current	negative	Oral cavity
**3**	female	65	50-69	former	negative	Oral cavity
**4**	male	49	<50	current	n.p.	Oral cavity
**5**	male	68	50-69	n.p.	n.p.	Oropharynx
**6**	male	63	50-69	current	n.p.	Oral cavity
**7**	female	44	<50	current	n.p.	Hypopharynx & Larynx
**8**	male	71	>69	current	n.p.	Hypopharynx & Larynx
**9**	male	63	50-69	former	negative	Oropharynx
**10**	male	51	50-69	current	negative	Oral cavity
**11**	female	70	>69	current	negative	Oropharynx
**12**	male	46	<50	current	negative	Oropharynx
**13**	male	67	50-69	never	positive	Oropharynx
**14**	male	72	>69	former	positive	Oropharynx
**15**	female	50	50-69	current	negative	Oral cavity
**16**	male	55	50-69	current	negative	Oropharynx
**17**	male	77	>69	n.p.	negative	Hypopharynx & Larynx
**18**	female	45	<50	current	negative	Oropharynx
**19**	male	68	50-69	former	positive	Oropharynx
**20**	male	58	50-69	former	negative	Oropharynx
**21**	male	66	50-69	current	negative	Oropharynx
**22**	male	58	50-69	former	positive	Oropharynx
**23**	female	47	<50	current	positive	Oropharynx
**24**	female	60	50-69	current	negative	Oral cavity
**25**	male	54	50-69	current	negative	Hypopharynx & Larynx
**26**	female	68	50-69	current	negative	Oropharynx
**27**	male	43	<50	current	n.p.	Oral cavity
**28**	female	46	<50	current	positive	Oropharynx
**29**	female	68	50-69	n.p.	n.p.	Hypopharynx & Larynx
**30**	male	42	<50	n.p.	n.p.	Oropharynx
**31**	female	77	>69	current	negative	Oral cavity
**32**	male	78	>69	never	positive	Oropharynx
**33**	male	49	<50	current	negative	Oral cavity
**34**	male	61	50-69	former	negative	Oral cavity
**35**	male	61	50-69	current	negative	Oral cavity
**36**	male	66	50-69	former	negative	Oropharynx
**37**	male	70	>69	current	negative	Oropharynx
**38**	male	74	>69	never	positive	Oropharynx
**39**	male	55	50-69	former	positive	Oropharynx
**40**	male	64	50-69	n.p.	n.p.	Hypopharynx & Larynx
**41**	male	56	50-69	current	negative	Oral cavity
**42**	male	52	50-69	former	negative	Oropharynx
**43**	female	54	50-69	former	negative	Oral cavity
**44**	female	78	>69	never	negative	Oral cavity
**45**	male	92	>69	former	positive	Oropharynx
**46**	male	70	>69	current	negative	Oral cavity
**47**	male	61	50-69	current	negative	Oropharynx
**48**	male	69	50-69	current	negative	Oropharynx
**49**	female	63	50-69	never	positive	Oropharynx
**50**	male	67	50-69	n.p.	negative	Hypopharynx & Larynx
**51**	male	71	>69	current	negative	Oropharynx
**52**	male	63	50-69	current	negative	Oral cavity
**53**	male	54	50-69	never	positive	Oropharynx

**54**	male	49	<50	never	positive	Oropharynx
**55**	male	50	50-69	n.p.	negative	Hypopharynx & Larynx
**56**	male	49	<50	former	positive	Oropharynx
**57**	male	78	>69	never	positive	Oropharynx
**58**	male	75	>69	never	positive	Oropharynx
**59**	male	77	>69	n.p.	n.p.	Oropharynx
**60**	male	62	50-69	current	negative	Oral cavity
**61**	male	50	50-69	n.p.	n.p.	Oropharynx
**62**	male	50	50-69	n.p.	n.p.	Oral cavity
**63**	male	51	50-69	former	n.p.	Hypopharynx & Larynx
**64**	male	79	>69	current	negative	Oral cavity
**65**	male	69	50-69	never	positive	Oropharynx
**66**	male	62	50-69	Never	positive	Oropharynx
**67**	male	54	50-69	current	negative	Oropharynx
**68**	male	65	50-69	Never	positive	Oropharynx
**69**	male	65	50-69	former	negative	Oropharynx
**70**	male	56	50-69	former	positive	Oropharynx
**71**	female	55	50-69	current	positive	Oropharynx
**72**	female	64	50-69	current	negative	Hypopharynx & Larynx
**73**	male	57	50-69	current	n.p.	Hypopharynx & Larynx
**74**	male	62	50-69	former	negative	Hypopharynx & Larynx
**75**	female	61	50-69	current	negative	Oropharynx
**76**	male	68	50-69	former	negative	Hypopharynx & Larynx
**77**	male	75	>69	former	n.p.	Hypopharynx & Larynx
**78**	male	68	50-69	Never	positive	Oropharynx
**79**	male	63	50-69	n.p.	n.p.	Oral cavity
**80**	male	62	50-69	current	negative	Oropharynx
**81**	male	44	<50	former	positive	Oropharynx
**82**	male	74	>69	current	negative	Hypopharynx & Larynx
**83**	female	71	>69	Never	negative	Oral cavity
**84**	female	52	50-69	current	negative	Oropharynx
**85**	male	48	<50	former	positive	Oropharynx
**86**	male	49	<50	current	negative	Oral cavity
**87**	female	49	<50	current	negative	Oropharynx
**88**	male	57	50-69	former	positive	Oropharynx
**89**	male	64	50-69	former	n.p.	Oral cavity
**90**	male	53	50-69	current	negative	Oropharynx
**91**	male	61	50-69	former	positive	Oral cavity
**92**	male	68	50-69	current	positive	Oropharynx
**93**	male	61	50-69	current	positive	Oropharynx
**94**	male	55	50-69	current	positive	Oropharynx

Patient	T Stage	N Stage
**1**	pT3	cN0
**2**	pT2	cN0
**3**	pT2	cN0
**4**	pT3	pN0
**5**	n.p.	n.p.
**6**	n.p.	cN0
**7**	pT3	pN1
**8**	pT3	cN0
**9**	pT3	cN0
**10**	pT2	pN2a
**11**	pT1	pN0
**12**	pT2	pN0
**13**	pT2	pN2c
**14**	pT2	pN1
**15**	pT3	pN2c
**16**	pT2	pN2c
**17**	pT1	cN0
**18**	pT3	pN2b
**19**	pT3	pN1
**20**	pT4a	pN3
**21**	pT2	pN0
**22**	pT3	pN3
**23**	pT3	pN1
**24**	pT1	pN0
**25**	pT4a	pN2b
**26**	pT3	pN0
**27**	pT1	pN0
**28**	pT2	pN2b
**29**	pT1	pN0
**30**	pT2	pN2b
**31**	pT2	pN0
**32**	pT3	pN2b
**33**	pT3	pN1
**34**	pT2	pN2b
**35**	pT3	pN2c
**36**	pT3	pN2b
**37**	pT2	pN2b
**38**	pT3	pN1
**39**	pT2	pN0
**40**	pT2	pN0
**41**	pT1	pN0
**42**	pT3	pN2c
**43**	pT3	pN1
**44**	pT2	pN0
**45**	pT3	pN1
**46**	pT2	pN1
**47**	pT3	pN0
**48**	pT3	pN2b
**49**	pT2	pN1
**50**	pT4b	pN1
**51**	pT3	pN2b
**52**	pT2	pN2c
**53**	pT2	pN2c
**54**	pT3	pN0
**55**	pT4a	pN2c

**56**	pT2	pN2a
**57**	pT3	pN2b
**58**	pT2	pN2c
**59**	pT2	pN2b
**60**	pT2	pN0
**61**	pT1	pN2b
**62**	pT2	pN2b
**63**	pT3	pN2a
**64**	pT2	pN0
**65**	pT2	pN2b
**66**	pT2	pN2b
**67**	pT3	pN2b
**68**	pT2	pN2a
**69**	pT1	pN2b
**70**	pT3	pN2b
**71**	pT1	pN2b
**72**	pT4a	pN2c
**73**	pT3	cN0
**74**	pT1	cN0
**75**	pT2	pN0
**76**	pT4a	pN1
**77**	pT2	pN1
**78**	pT1	pN1
**79**	pT1	pN0
**80**	pT4a	pN1
**81**	pT2	pN2b
**82**	pT3	pN0
**83**	pT3	pN0
**84**	pT3	cN0
**85**	pT2	pN2b
**86**	pT2	pN0
**87**	pT3	pN2b
**88**	pT2	pN0
**89**	pT2	pN0
**90**	pT3	pN3
**91**	pT1	pN1
**92**	pT2	pN1
**93**	pT3	pN2b
**94**	pT2	pN2c

Supplementary Table 2: IHC scores of EpEX, Sox2, and vimentin expression in HNSCC primary tumors of the Göttingen cohort (n=94). Scores have been evaluated by two experimenters and are shown separately as well as mean IHC scores. Data on gender, age, smoking behavior, p16 expression as a surrogate for HPV status, HNSCC tumor localization as well as TN status and whether chemotherapy was administered together with primary radiation are listed beneath.

Patient	EpEx IHC score 1	EpEx % x int.	EpEx IHC score 2	EpEx % x int.	Mean EpEx IHC score
**1**	0	100*0	0	100*0	0
**2**	0	100*0	0	100*0	0
**3**	0	100*0	0	100*0	0
**4**	0	100*0			0
**5**	240	80*3 20*0	240	80*3 20*0	240
**6**	30	10*3 90*0	60	20*3 80*0	45
**7**	150	50*3 50*0	150	50*3 50*0	150
**8**	150	50*3 50*0	150	50*3 50*0	150
**9**	200	100*2			200
**10**	150	50*3 50*0	150	50*3 50*0	150
**11**	300	100*3			300
**12**	150	50*3 50*0	150	50*3 50*0	150
**13**	150	50*3 50*0			150
**14**	0	100*0			0
**15**	60	20*3 80*0			60
**16**	240	80*3 20*0			240
**17**	0	100*0			0
**18**	5	95*0			5
**19**	200	100*2	160	80*2 20*0	180
**20**	300	100*3	300	100*3	300
**21**	0	100*0			0
**22**	80	40*2 60*0	40	20*2 80*0	60
**23**	60	30*2 70*0	40	20*2 80*0	50
**24**	300	100*3	300	100*3	300
**25**	0	100*0	0	100*0	0
**26**	150	50*3 50*0	150	50*3 50*0	150
**27**	120	40*3 60*0	0	100*0	60
**28**	120	60*2 40*0	120	60*2 40*0	120
**29**	0	100*0	0	100*0	0
**30**	30	10*3 90*0	120	40*3 60*0	75
**31**	90	30*3 70*0	90	30*3 70*0	90
**32**	120	40*3 60*0	120	40*3 60*0	120
**33**	160	80*2 20*0	160	80*2 20*0	160
**34**	0	100*0	0	100*0	0
**35**	300	100*3	300	100*3	300
**36**	0	100*0	0	100*0	0
**37**	15	5*3 95*0	15	5*3 95*0	15
**38**	30	10*3 90*0	30	10*3 90*0	30
**39**	0	100*0			0
**40**					
**41**	90	30*3 70*0	90	30*3 70*0	90
**42**	90	30*3 70*0			90
**43**	90	30*3 70*0	90	30*3 70*0	90
**44**	0	100*0	0	100*0	0
**45**	0	100*0	0	100*0	0
**46**	0	100*0	0	100*0	0
**47**					
**48**	300	100*3			300
**49**	0	100*0			0

**50**	0	100*0	0	100*0	0
**51**	0	100*0			0
**52**	300	100*3	300	100*3	600
**53**	0	100*0	0	100*0	0
**54**	300	100*3	300	100*3	300
**55**	0	100*0	0	100*0	0
**56**	0	100*0	60	20*3 80*0	60
**57**	30	10*3 90*0			30
**58**					
**59**	240	80*3 20*0	270	90*3 10*0	255
**60**	0	100*0			0
**61**					
**62**	60	20*3 80*0	60	20*3 80*0	60
**63**	0	100*0	0	100*0	0
**64**	150	50*3 50*0			150
**65**	0	100*0	0	100*0	0
**66**					
**67**					
**68**					
**69**	0	100*0			0
**70**	300	100*3	300	100*3	300
**71**	0	100*0	0	100*0	0
**72**	60	20*3 80*0	30	10*3 90*0	45
**73**	180	60*3 40*0	180	60*3 40*0	180
**74**	0	100*0	0	100*0	0
**75**	180	60*3 40*0	180	60*3 40*0	180
**76**	0	100*0	0	100*0	0
**77**	240	80*3 20*0	240	80*3 20*0	240
**78**	0	100*0	0	100*0	0
**79**	0	100*0	0	100*0	0
**80**	15	5*3 95*0			15
**81**	0	100*0	0	100*0	0
**82**	15	5*3 95*0			15
**83**	90	30*3 70*0	90	30*3 70*0	90
**84**	0	100*0	0	100*0	0
**85**	0	100*0			0
**86**	300	100*3	300	100*3	300
**87**	300	100*3	300	100*3	300
**88**	90	30*3 70*0	90	30*3 70*0	90
**89**	300	100*3	300	100*3	300
**90**	0	100*0	0	100*0	0
**91**	0	100*0	0	100*0	0
**92**	0	100*0	0	100*0	0
**93**	0	100*0	0	100*0	0
**94**	0	100*0	0	100*0	0

**Patient**	**Sox2 IHC score 1**	**Sox2 % x int.**	**Sox2 IHC score 2**	**Sox2 % x int.**	**Mean Sox2 IHC score**
**1**	0	100*0	0	100*0	0
**2**	0	100*0	50	50*1 50*0	25
**3**	100	50*2 50*0	100	50*2 50*0	100
**4**	0	100*0	300	100*3	150
**5**			300	100*3	300

**6**			10	10*1 90*0	10
**7**	200	100*2	300	100*3	250
**8**	0		0		0
**9**	100	50*2 50*0	100	50*2 50*0	100
**10**	300	100*3	300	100*3	300
**11**			300	100*3	300
**12**	250	50*3 50*2	0	100*0	125
**13**	130	20*3 30*2 10*1 40*0			130
**14**	10	10*1 90*0	30	10*2 10*1 80*0	20
**15**	300	100*3			300
**16**	40	10*2 20*1 70*0	0		20
**17**	0	100*0			0
**18**	0		0		0
**19**	160	80*2 20*0	140	70*2 30*0	150
**20**	300	100*3	300	100*3	300
**21**					
**22**	170	30*3 40*2 30*0	200	100*2	185
**23**	250	50*3 50*2	250	50*3 50*2	250
**24**			40	20*2 80*0	40
**25**	300	100*3	300	100*3	300
**26**	200	100*2	200	100*2	200
**27**	0	100*0	0	100*0	0
**28**	20	20*1 80*0	20	20*1 80*0	20
**29**	300	100*3			300
**30**	160	80*2 20*0	40	20*2 80*0	100
**31**	0	100*0	0	100*0	0
**32**	200	100*2	200	100*2	200
**33**	300	100*3	300	100*3	300
**34**	0	100*0	0	100*0	0
**35**	300	100*3	300	100*3	300
**36**	40	20*2 80*0	100	50*2 50*0	70
**37**	80	40*2 60*0	80	40*2 60*0	80
**38**	300	100*3	150	50*2 50*1	225
**39**	120	60*2 40*0	0		60
**40**	210	70*3 30*0	110	30*3 20*1 50*0	160
**41**	0	100*0	0	100*0	0
**42**	10	10*1 90*0	15	5*2 5*1 90*0	12.5
**43**	300	100*3	300	100*3	300
**44**	0	100*0	0	100*0	0
**45**	300	100*3	300	100*3	300
**46**	190	50*3 20*2 30*0	160	80*2 20*0	175
**47**			0	100*0	0
**48**	20	10*2 90*0	20	10*2 90*0	20
**49**					
**50**	200	100*2	100	50*2 50*0	150
**51**	40	20*2 80*0	40	20*2 80*0	40
**52**	300	100*3	300	100*3	300
**53**	80	30*2 20*1 50*0	80	30*2 20*1 50*0	80
**54**	120	60*2 40*0	40	20*2 80*0	80
**55**	20	20*1 80*0	20	20*1 80*0	20
**56**	0	100*0	250	50*3 50*2	125
**57**	160	60*2 40*1			160
**58**	0	100*0	0	100*0	0
**59**	0	100*0	0	100*0	0
**60**			0	100*0	0
**61**	0	100*0			0

**62**	300	100*3	300	100*3	300
**63**	0	100*0	40	20*2 80*0	20
**64**	120	20*2 80*1			120
**65**	100	20*3 20*2 60*0			100
**66**	0	100*0	0	100*0	0
**67**	0	100*0			0
**68**	300	100*3	300	100*3	300
**69**			0	100*0	0
**70**	200	100*2	200	100*2	200
**71**	0	100*0	60	30*2 70*0	30
**72**	300	100*3	300	100*3	300
**73**	210	50*3 30*2 20*0	280	80*3 20*2	245
**74**	200	100*2	200	100*2	200
**75**	0	100*0	0	100*0	0
**76**	40	20*2 80*0	0	100*0	20
**77**	200	100*2	200	100*2	200
**78**	0	100*0	10	10*1 90*0	5
**79**	0	100*0	0	100*0	0
**80**	30	10*2 10*1 80*0			30
**81**	0	100*0	0	100*0	0
**82**	0	100*0	10	10*1 90*0	5
**83**	25	5*3 5*2 90*0	0	100*0	12.5
**84**	300	100*3	300	100*3	300
**85**			180	80*2 20*1	180
**86**	300	100*3	300	100*3	300
**87**	180	80*2 20*1	180	80*2 20*1	180
**88**	0	100*0	70	20*2 30*1 50*0	35
**89**	0	100*0	200	100*2	100
**90**	30	10*2 10*1 80*0	30	10*2 10*1 80*0	30
**91**	0	100*0	0	100*0	0
**92**			0	100*0	0
**93**	0	100*0	0	100*0	0
**94**	0	100*0	0	100*0	0

**Patient**	**Vimentin IHC score 1**	**Vimentin % x int.**	**Vimentin ICH score 2**	**Vimentin % x int.**	**Mean Vimentin score**
**1**	0	100*0	0	100*0	0
**2**	0	100*0	0	100*0	0
**3**	60	20*3 80*0	60	20*3 80*0	60
**4**	0	100*0	0	100*0	0
**5**	0	100*0	0	100*0	0
**6**			0	100*0	0
**7**	0	100*0	0	100*0	0
**8**	0	100*0			0
**9**			60	20*3 80*0	60
**10**	0	100*0	0	100*0	0
**11**			0	100*0	0
**12**	0	100*0	0	100*0	0
**13**	240	80*3 20*0			240
**14**			0	100*0	0
**15**	0	100*0			0
**16**	0	100*0			0

**17**	0	100*0	0	100*0	0
**18**	0	100*0			0
**19**	60	20*3 80*0	60	20*3 80*0	60
**20**	300	100*3	300	100*3	300
**21**	300	100*3			300
**22**	0	100*0	0	100*0	0
**23**	0	100*0	0	100*0	0
**24**	0	100*0	0	100*0	0
**25**	60	20*3 80*0	60	20*3 80*0	60
**26**	60	20*3 80*0	210	70*3 30*0	135
**27**	0	100*0			0
**28**	60	20*3 80*0	60	20*3 80*0	60
**29**					
**30**	0	100*0	60	20*3 80*0	30
**31**	0	100*0	0	100*0	0
**32**	0	100*0	0	100*0	0
**33**	0	100*0	0	100*0	0
**34**	150	50*3 50*0	150	50*3 50*0	150
**35**	0	100*0	0	100*0	0
**36**			0	100*0	0
**37**	300	100*3	300	100*3	300
**38**	0	100*0	0	100*0	0
**39**					
**40**	0	100*0	0	100*0	0
**41**	0	100*0	0	100*0	0
**42**	0	100*0	0	100*0	0
**43**	0	100*0	0	100*0	0
**44**	240	80*3 20*0	240	80*3 20*0	240
**45**			0	100*0	0
**46**	150	50*3 50*0	150	50*3 50*0	150
**47**					
**48**	0	100*0	0	100*0	0
**49**					
**50**	0	100*0	0	100*0	0
**51**					
**52**	0	100*0			0
**53**	90	30*3 70*0	150	50*3 50*0	120
**54**	240	80*3 20*0	240	80*3 20*0	240
**55**	0	100*0	0	100*0	0
**56**	0	100*0	0	100*0	0
**57**	0	100*0	0	100*0	0
**58**					
**59**	240	80*3 20*0	240	80*3 20*0	240
**60**					
**61**					
**62**	0	100*0	0	100*0	0
**63**	60	20*3 80*0	60	20*3 80*0	60
**64**	0	100*0			0
**65**			0	100*0	0
**66**					
**67**					
**68**	0	100*0	0	100*0	0
**69**	0	100*0			0
**70**	0	100*0	0	100*0	0
**71**	90	30*3 70*0	90	30*3 70*0	90
**72**	150	50*3 50*0	60	20*3 80*0	105

**73**	0	100*0	0	100*0	0
**74**	0	100*0	0	100*0	0
**75**	0	100*0	0	100*0	0
**76**	0	100*0	0	100*0	0
**77**					
**78**			0	100*0	0
**79**	0	100*0	0	100*0	0
**80**	150	50*3 50*0			150
**81**	0	100*0	0	100*0	0
**82**	0	100*0			0
**83**	0	100*0	0	100*0	0
**84**	0	100*0	0	100*0	0
**85**	0	100*0			0
**86**	0	100*0	0	100*0	0
**87**	0	100*0	0	100*0	0
**88**	240	80*3 20*0	240	80*3 20*0	240
**89**			0	100*0	0
**90**	0	100*0	0	100*0	0
**91**	0	100*0	0	100*0	0
**92**	0	100*0	0	100*0	0
**93**	0	100*0	0	100*0	0
**94**			300	100*3	300

Patient	Gender	Age	Age groups	Smoking status	p16	Localisation
**1**	male	53.74	50-69	ever	positive	Hypopharynx & Larynx
**2**	male	54.83	50-69	ever	positive	Oral cavity
**3**	female	55.77	50-69	ever	negative	Oropharynx
**4**	male	77.54	>69	never	negative	Hypopharynx & Larynx
**5**	male	69.65	50-69	ever	positive	Oropharynx
**6**	male	46.21	<50	never	positive	Oral cavity
**7**	female	60.12	50-69	ever	negative	Oropharynx
**8**	male	70.67	>69	ever	positive	Oral cavity
**9**	male	66.67	50-69	ever	negative	Oropharynx
**10**	male	82.40	>69	ever	positive	Oral cavity
**11**	male	52.56	50-69	never	negative	Oropharynx
**12**	male	51.04	50-69	ever	positive	Oropharynx
**13**	male	56.00	50-69	ever	positive	Oropharynx
**14**	male	58.78	50-69	ever	positive	Hypopharynx & Larynx
**15**	male	64.12	50-69	ever	negative	Oropharynx
**16**	male	68.62	50-69	ever	positive	Oral cavity
**17**	male	78.93	>69	never	negative	Oropharynx
**18**	female	81.33	>69	never	negative	Oral cavity
**19**	male	58.82	50-69	ever	positive	Hypopharynx & Larynx
**20**	male	68.88	50-69	never	positive	Oropharynx
**21**	male	63.19	50-69	ever	positive	Oropharynx
**22**	male	55.72	50-69	never	negative	Hypopharynx & Larynx
**23**	male	62.90	50-69	never	positive	Oral cavity
**24**	male	63.78	50-69	ever	positive	Hypopharynx & Larynx
**25**	male	65.43	50-69	never	positive	Oropharynx
**26**	male	52.31	50-69	ever	positive	Oropharynx
**27**	male	55.98	50-69	ever	negative	Hypopharynx & Larynx
**28**	male	59.92	50-69	ever	negative	Oropharynx
**29**	male	47.94	<50	ever	positive	Oropharynx
**30**	male	53.68	50-69	ever	positive	Hypopharynx & Larynx
**31**	male	46.00	<50	ever	positive	Oropharynx
**32**	male	60.18	50-69	ever	positive	Hypopharynx & Larynx
**33**	male	49.86	<50	ever	positive	Oropharynx
**34**	male	62.39	50-69	ever	negative	Oral cavity
**35**	male	56.58	50-69	ever	negative	Oropharynx
**36**	male	60.85	50-69	ever	negative	Hypopharynx & Larynx
**37**	male	68.59	50-69	ever	positive	Oral cavity
**38**	male	57.89	50-69	ever	negative	Hypopharynx & Larynx
**39**	male	48.00	<50	ever	negative	Oral cavity
**40**	male	55.32	50-69	ever	positive	Hypopharynx & Larynx
**41**	male	58.20	50-69	ever	negative	Oropharynx
**42**	male	45.88	<50	never	negative	Oropharynx
**43**	female	55.41	50-69	ever	positive	Oral cavity
**44**	male	65.62	50-69	ever	negative	Hypopharynx & Larynx
**45**	male	40.50	<50	ever	negative	Oropharynx
**46**	male	50.05	50-69	ever	positive	Oropharynx
**47**	female	43.79	<50	never	negative	Oral cavity
**48**	male	65.47	50-69	ever	negative	Oral cavity
**49**	male	84.24	>69	ever	positive	Oropharynx
**50**	male	65.01	50-69	ever	positive	Oral cavity
**51**	female	41.96	<50	ever	negative	Oral cavity
**52**	male	53.45	50-69	ever	negative	Oropharynx
**53**	female	70.71	>69	ever	positive	Oropharynx
**54**	male	44.39	<50	ever	negative	Oral cavity

**55**	male	50.19	50-69	ever	negative	Oral cavity
**56**	male	63.16	50-69	never	negative	Hypopharynx & Larynx
**57**	male	61.19	50-69	never	negative	Hypopharynx & Larynx
**58**	male	50.96	50-69	ever	positive	Hypopharynx & Larynx
**59**	male	52.59	50-69	never	negative	Hypopharynx & Larynx
**60**	male	67.17	50-69	ever	negative	Oropharynx
**61**	male	64.79	50-69	ever	negative	Oropharynx
**62**	male	59.25	50-69	ever	positive	Hypopharynx & Larynx
**63**	male	57.59	50-69	ever	negative	Hypopharynx & Larynx
**64**	male	44.02	<50	ever	positive	Oropharynx
**65**	male	56.64	50-69	ever	positive	Oropharynx
**66**	male	48.05	<50	ever	negative	Hypopharynx & Larynx
**67**	male	60.52	50-69	ever	positive	Oral cavity
**68**	male	51.92	50-69	ever	negative	Oropharynx
**69**	female	20.68	<50	ever	negative	Oral cavity
**70**	male	49.64	<50	ever	positive	Hypopharynx & Larynx
**71**	male	48.93	<50	ever	negative	Oral cavity
**72**	male	52.84	50-69	ever	negative	Oropharynx
**73**	male	57.06	50-69	ever	positive	Hypopharynx & Larynx
**74**	female	54.80	50-69	ever	negative	Oral cavity
**75**	male	49.80	<50	ever	negative	Oropharynx
**76**	male	61.99	50-69	ever	negative	Hypopharynx & Larynx
**77**	female	55.00	50-69	ever	negative	Hypopharynx & Larynx
**78**	female	82.06	>69	ever	positive	Oral cavity
**79**	female	88.77	>69	never	positive	Oral cavity
**80**	male	53.66	50-69	ever	negative	Oral cavity
**81**	male	70.99	>69	ever	positive	Oral cavity
**82**	male	45.08	<50	ever	negative	Oral cavity
**83**	female	64.60	50-69	never	positive	Oropharynx
**84**	female	49.45	<50	ever	positive	Oropharynx
**85**	male	53.90	50-69	ever	negative	Oropharynx
**86**	male	48.92	<50	ever	positive	Oropharynx
**87**	female	70.72	>69	never	positive	Hypopharynx & Larynx
**88**	female	66.27	50-69	ever	negative	Oral cavity
**89**	male	56.14	50-69	ever	negative	Oropharynx
**90**	male	63.82	50-69	never	negative	Oral cavity
**91**	male	45.37	<50	ever	negative	Oral cavity
**92**	male	54.88	50-69	ever	positive	Oropharynx
**93**	male	69.71	50-69	ever	positive	Oral cavity
**94**	male	69.22	50-69	ever	negative	Hypopharynx & Larynx

Patient	T Stage	N Stage	Chemotherapy
1	pT2	pN2c	yes
2	pT4a/b	pN2a	yes
3	pT4a/b	pN0	no
4	pT4a/b	pN2b	no
5	pT4a/b	pN2b	no
6	pT4a/b	pN2b	yes
7	pT2	pN2c	yes
8	pT2	pN0	no
9	pT3	pN2b	yes
10	pT4a/b	pN2a	yes
11	pT4a/b	pN1	no
12	pT4a/b	pN2c	yes
13	pT4a/b	pN0	no
14	pT4a/b	pN0	yes
15	pT4a/b	pN2b	yes
16	pT4a/b	pN2b	yes
17	pT4a/b	pN2c	no
18	pT4a/b	pN2c	no
19	pT4a/b	pN3	no
20	pT4a/b	pN2b	yes
21	pT4a/b	pN2c	yes
22	pT4a/b	pN2c	yes
23	pT2	pN0	yes
24	pT4a/b	pN2b	yes
25	pT1	pN3	yes
26	pT4a/b	pN2c	yes
27	pT4a/b	pN2c	yes
28	pT4a/b	pN2c	yes
29	pT4a/b	pN3	yes
30	pT4a/b	pN2c	yes
31	pT4a/b	pN2a	yes
32	pT4a/b	pN2c	yes
33	pT4a/b	pN2b	no
34	pT4a/b	pN2a	yes
35	pT4a/b	pN2c	yes
36	pT1	pN2b	yes
37	pT2	pN0	yes
38	pT4a/b	pN2c	yes
39	pT4a/b	pN0	yes
40	pT4a/b	pN2a	yes
41	pT4a/b	pN2c	yes
42	pT4a/b	pN1	no
43	pT3	pN2a	yes
44	pT4a/b	pN0	yes
45	pT4a/b	pN2c	yes
46	pT4a/b	pN2b	yes
47	pT4a/b	pN0	yes
48	pT4a/b	pN2a	yes
49	pT4a/b	pN2b	yes
50	pT2	pN0	yes
51	pT4a/b	pN2b	yes
52	pT3	pN2c	yes
53	pT4a/b	pN2c	yes
54	pT4a/b	pN1	yes
55	pT4a/b	pN2c	yes

**56**	pT3	pN2b	yes
**57**	pT4a/b	pN3	yes
**58**	pT4a/b	pN2c	yes
**59**	pT4a/b	pN2a	yes
**60**	pT2	pN1	yes
**61**	pT4a/b	pN3	yes
**62**	pT3	pN2c	yes
**63**	pT4a/b	pN2c	no
**64**	pT4a/b	pN2c	no
**65**	pT4a/b	pN0	yes
**66**	pT4a/b	pN3	yes
**67**	pT3	pN1	yes
**68**	pT4a/b	pN2c	no
**69**	pT1	pN2a	yes
**70**	pT3	pN2b	yes
**71**	pT4a/b	pN2c	yes
**72**	pT1	pN1	yes
**73**	pT4a/b	pN2c	no
**74**	pT3	pN0	yes
**75**	pT4a/b	pN2b	yes
**76**	pT3	pN0	yes
**77**	pT4a/b	pN2b	yes
**78**	pT3	pN2c	no
**79**	pT4a/b	pN2a	no
**80**	pT4a/b	pN2b	yes
**81**	pT3	pN0	no
**82**	pT4a/b	pN2c	yes
**83**	pT4a/b	pN1	yes
**84**	pT4a/b	pN2b	yes
**85**	pT4a/b	pN2a	no
**86**	pT4a/b	pN1	yes
**87**	pT4a/b	pN2c	yes
**88**	pT4a/b	pN2c	yes
**89**	pT4a/b	pN2a	yes
**90**	pT3	pN1	yes
**91**	pT4a/b	pN1	yes
**92**	pT4a/b	pN2c	yes
**93**	pT3	pN0	yes
**94**	pT2	pN0	yes
